# Constitutive Defense Mechanisms Have a Major Role in the Resistance of Woodland Strawberry Leaves Against *Botrytis cinerea*

**DOI:** 10.3389/fpls.2022.912667

**Published:** 2022-07-06

**Authors:** Yijie Zhao, Liese Vlasselaer, Bianca Ribeiro, Konstantinos Terzoudis, Wim Van den Ende, Maarten Hertog, Bart Nicolaï, Barbara De Coninck

**Affiliations:** ^1^Division of Crop Biotechnics, Department of Biosystems, KU Leuven, Leuven, Belgium; ^2^Division of Mechatronics, Biostatistics and Sensors, Department of Biosystems, KU Leuven, Leuven, Belgium; ^3^KU Leuven Plant Institute, Heverlee, Belgium; ^4^Laboratory of Molecular Plant Biology, Department of Biology, KU Leuven, Leuven, Belgium; ^5^Flanders Centre of Postharvest Technology, Leuven, Belgium

**Keywords:** woodland strawberry, *Botrytis cinerea*, defense-related genes, hydrogen peroxide, antioxidant enzymes, ascorbic acid, specialized metabolites, primary metabolites

## Abstract

The necrotrophic fungus *Botrytis cinerea* is a major threat to strawberry cultivation worldwide. By screening different *Fragaria vesca* genotypes for susceptibility to *B. cinerea*, we identified two genotypes with different resistance levels, a susceptible genotype *F. vesca* ssp. *vesca* Tenno 3 (T3) and a moderately resistant genotype *F. vesca* ssp. *vesca* Kreuzkogel 1 (K1). These two genotypes were used to identify the molecular basis for the increased resistance of K1 compared to T3. Fungal DNA quantification and microscopic observation of fungal growth in woodland strawberry leaves confirmed that the growth of *B. cinerea* was restricted during early stages of infection in K1 compared to T3. Gene expression analysis in both genotypes upon *B. cinerea* inoculation suggested that the restricted growth of *B. cinerea* was rather due to the constitutive resistance mechanisms of K1 instead of the induction of defense responses. Furthermore, we observed that the amount of total phenolics, total flavonoids, glucose, galactose, citric acid and ascorbic acid correlated positively with higher resistance, while H_2_O_2_ and sucrose correlated negatively. Therefore, we propose that K1 leaves are more resistant against *B. cinerea* compared to T3 leaves, prior to *B. cinerea* inoculation, due to a lower amount of innate H_2_O_2_, which is attributed to a higher level of antioxidants and antioxidant enzymes in K1. To conclude, this study provides important insights into the resistance mechanisms against *B. cinerea*, which highly depend on the innate antioxidative profile and specialized metabolites of woodland strawberry leaves.

## Introduction

Strawberry (*Fragaria × ananassa*) is one of the most important berry crops worldwide with a global production of approximately 8.9 million metric tons in 2019 (FAOSTAT, 2021). The fruit are highly appreciated for their flavor and nutritional values (Ulrich et al., 1997; Wang and Lin, 2000; Giampieri et al., 2015). However, strawberry cultivation is often hampered by the occurrence of numerous pests and diseases, including the necrotrophic fungus *Botrytis cinerea*, resulting in gray mold of fruit and leaves and, as such, causing huge production and economic losses (Petrasch et al., 2019). *B. cinerea* is mainly controlled by fungicides. However, the adverse effects of fungicide use on the environment and the development of fungicide-resistant strains urge the development of alternative methods to limit *B. cinerea* infection of strawberry. Breeding for more resistant varieties is a promising process, but it is not straightforward as resistance in strawberry to *B. cinerea* is quantitative, genetically complex and depends on a wide variety of defense mechanisms. To achieve this goal, a detailed comparative study of *B. cinerea*-susceptible and resistant cultivars is needed to better understand the underlying mechanisms of increased resistance against *B. cinerea*.

*B. cinerea* produces cell wall degrading enzymes, reactive oxygen species (ROS), and toxins to infect and colonize plants (Blanco-Ulate et al., 2013; Nakajima and Akutsu, 2014). Likewise, plants also react *via* different mechanisms to restrict *B. cinerea* infection, for example the induction of hormonal signaling pathways regulating the production of defense-related proteins and specialized metabolites, and the production of reactive oxygen species (ROS). Also constitutive levels of defense-related molecules prior to pathogen attack can be critical for the outcome of the interaction. Upon *B. cinerea* inoculation of strawberry fruit, genes involved in ethylene (ET) and jasmonic acid (JA) biosynthesis and signaling are upregulated including *1-AMINOCYCLOPROPANE-1-CARBOXYLATE SYNTHASE 2* (*ACS2*)*, 1-AMINOCYCLOPROPANE-1-CARBOXYLATE OXYGENASE* (*ACO*)*, LIPOXYGENASE* (*LOX*) and *ALLENE OXIDE SYNTHASE* (*AOS*; Xiong et al., 2018; Haile et al., 2019). Additionally, the activity and expression of pathogenesis-related proteins, such as *β*-1,3-glucanases (βGLU) and chitinases, pathogenesis related protein family 10 (PR10) and polygalacturonase inhibitor proteins (PGIPs), enzymes inhibiting polygalacturonases produced by pathogens, are upregulated in strawberry fruit after *B. cinerea* inoculation (Mehli et al., 2004, 2005; Nagpala et al., 2016; Wang et al., 2016; Haile et al., 2019). Interestingly, the constitutive level of PGIPs, prior to *B. cinerea* inoculation, also contributes to strawberry fruit resistance. For example, a study on five *F. × ananassa* cultivars demonstrated that the cultivar with the highest constitutive expression level of *PGIP* was the least susceptible to *B. cinerea* (Mehli et al., 2004). Moreover, overexpression of *FaPGIP* in strawberry plants resulted in increased resistance against *B. cinerea* (Schaart, 2004).

Second, the production of ROS by plant cells is an important strategy against biotic stresses (Jajic et al., 2015). Hydrogen peroxide (H_2_O_2_), one of the most important ROS compounds, can either directly inhibit pathogen growth or act as a signaling molecule leading to reinforcement of cell walls, accumulation of phytoalexins, and programmed cell death (PCD), specifically restricting growth of biotrophic pathogens (Kärkönen and Kuchitsu, 2015; Huang et al., 2019). However, the production of ROS, leading to host cell death, facilitates infection by necrotrophic pathogens such as *B. cinerea* (Govrin and Levine, 2000; Khanam et al., 2005). Interestingly, constitutively higher levels of H_2_O_2_ and O_2_^−^ correlated both negatively as well as positively with plant resistance against several biotic stresses (Asselbergh et al., 2007; Meng et al., 2019; Shao et al., 2019; Rahman et al., 2020). In both grapevine and strawberry, it has been reported that increased resistance against *B. cinerea* is linked with low basal levels of ROS (H_2_O_2_ and O_2_^−^; Meng et al., 2019; Rahman et al., 2020). The delicate balance between ROS production and scavenging is also important for plant survival under adverse conditions. Plants contain enzymatic and non-enzymatic antioxidant systems essential for ROS homeostasis (Gill and Tuteja, 2010a). Enzymatic systems include superoxide dismutase (SOD), catalase (CAT), ascorbate peroxidase (APX), glutathione peroxidase (GPX), glutathione reductase (GR) and glutathione S-transferase (GST) while non-enzymatic compounds include ascorbic acid (AsA), phenolics, flavonoids, glutathione and sugars (Gill and Tuteja, 2010b; Das and Roychoudhury, 2014; Matros et al., 2015). The activity of antioxidant enzymes has also been suggested to correlate with plant resistance. For example, grapevine genotypes with increased resistance against *B. cinerea* showed higher constitutive peroxidase activity in leaves and fruit than susceptible genotypes, prior to inoculation (Rahman et al., 2020). A similar observation was made in tomato leaves, where SOD activity was higher in more resistant genotypes after *Alternaria solani* inoculation (Ray et al., 2015). AsA, one of the important non-enzymatic antioxidants known for its ability to scavenge H_2_O_2_ in plants either spontaneously or *via* APX enzymes, can improve stress tolerance in plants (Akram et al., 2017). AsA can affect plant resistance by the orchestration of different mechanisms. For example, in *Arabidopsis thaliana*, AsA-deficient mutants show increased PCD, an increased amount of salicylic acid and PR proteins leading to enhanced constitutive resistance against the hemibiotrophic pathogen *Pseudomonas syringae* (Pavet et al., 2005; Mukherjee et al., 2010). On the other hand, AsA-deficient mutants are less resistant to the necrotrophic pathogen *Alternaria brassicicola* (Botanga et al., 2012). Additionally, treatment of strawberry fruit with AsA inhibited *B. cinerea* growth, potentially by increasing the total phenolics content of strawberry (Elkorany and Mohamed, 2008). Similar to H_2_O_2_, AsA potentially influence plant disease resistance levels depending on the pathogen’s lifestyle.

Finally, both primary and specialized metabolites contribute to restricting pathogen infection either by having a role in plant resistance or by directly inhibiting the pathogen (Rojas et al., 2014). For example, spermine treatment resulted in higher levels of glucose, fructose and sucrose in Arabidopsis, as well as higher resistance to *B. cinerea*. Moreover, *Colletotrichum gloeosporioides* showed enhanced colonization in tomato fruit with low sugar content (Ziv et al., 2020). Acetic acid suppresses the growth of *C. gloeosporioides* directly, and lowers the rate of infection in strawberries (Kang et al., 2003; Weber et al., 2016). Strawberry fruit containing high levels of specialized metabolites are shown to have increased resistance against *B. cinerea*. Unripe strawberry fruit contain more flavonoids than mature strawberry fruit, including catechin, agrimoniin, ellagic acid conjugates, proanthocyanidins, and flavan-3-ols (Di Venere et al., 1998; Puhl and Treutter, 2008; Aaby et al., 2012), which could be one of the reasons why immature strawberry fruit is less sensitive to *B. cinerea*. Moreover, the concentration of proanthocyanidin has been used as a biochemical marker of strawberry fruit resistance against *B. cinerea* (Jersch et al., 1988; Di Venere et al., 1998; Hébert et al., 2002). Treatment of strawberry fruit with terpinen-4-ol improved plant resistance against *B. cinerea* by activating the phenylpropanoid metabolic pathway (Li et al., 2020). Similarly, treatment of strawberry leaves with red light or chitosan resulted in increased concentrations of total phenolics and flavonoids and higher resistance against *B. cinerea* (Meng et al., 2019; Peian et al., 2021).

The induced defense response of strawberry leaves and fruit against *B. cinerea* has been well-documented (Jersch et al., 1988; Nagpala et al., 2016; Bui et al., 2019; Haile et al., 2019; Hu et al., 2019; Petrasch et al., 2019). However, while it is clear that constitutive resistance mechanisms also play a role in the resistance of strawberry against *B. cinerea*, a comprehensive view on the underlying mechanisms is lacking. In the present study, we identified two woodland strawberry genotypes with altered resistance against *B. cinerea* and investigated the mechanisms behind the increased resistance of K1 compared to T3 by examining expression of defense-related genes, the production of H_2_O_2_, AsA, specialized and primary metabolites and the expression and activity of ROS scavenging enzymes, prior to inoculation.

## Materials and Methods

### Plant Material and Pathogen Inoculum

*Fragaria vesca* ssp. *vesca* Kreuzkogel 1 (K1) and *F. vesca* ssp. *vesca* Tenno 3 (T3; provided by “Professor Staudt Collection”) were grown in a greenhouse at 21–23°C under light (on average 14 h light/10 h dark, 250 W/m^2^) and a relative humidity of 65%, except for 1 h before sunset, the humidity was increased to 90%.

*Botrytis cinerea* strain B05.10 was cultivated on potato dextrose broth agar (PDA) medium for 5 d in the dark at 25°C, then exposed to UV-A (315 nm-400 nm) for 12 h and allowed to sporulate for 5–9 d in the dark. The spores were collected and the concentration was adjusted to 10^8^ spores/mL using a hemocytometer counting chamber and stored in 25% glycerol at −80°C. For leaf inoculations, *B. cinerea* spores were diluted to 10^6^ spores/mL with ½ potato dextrose broth (PDB).

### Leaf Inoculations and Disease Assays

During strawberry growth, leaves were labeled using their date of emergence. For all experiments, trifoliate strawberry leaves were collected, cut into three leaflets, disinfected with 5% bleach (Loda Bleach 10°) for 5 min and cleansed with sterile distilled water. To evaluate the susceptibility against *B. cinerea* of the two genotypes, 3 weeks old leaves were used. After disinfection, dried detached leaflets were immediately transferred to 0.8% agar plates and drop-inoculated with 5 μl of 10^6^ spores/mL on the adaxial leaf surface avoiding inoculation on the main vein. The plates were kept at room environment. For disease assays, the lesion area was measured 5 d post inoculation (dpi) and 14 or 15 detached leaflets from each genotype were evaluated. Wilcox test was used to evaluate statistically significant differences between the genotypes.

The resistance level of K1 and T3 genotypes was assessed based on the disease severity index (DSI) according to Curvers et al. (2010) and Rahman et al. (2018) with minor modifications. DSI was calculated using five different classes (0: no symptoms; 1: 0 < lesion area percentage < 25%; 2: 25% < lesion area percentage < 50%; 3: 50% < lesion area percentage < 75%; 4: 75% < lesion area percentage < 100%) using the formula [(0 × a) + (1 × b) + (2 × c) + (3 × d)/(a + b + c + d)] × 100/3 where a, b, c, and d are the number of leaves within the scores of 0, 1, 2, 3 or 4, respectively. Next, the DSI was used to classify the different resistance levels: 0: highly resistant; 0–25%: resistant; 25–50%: moderately resistant; 50–75%: susceptible; 75–100% highly susceptible.

To follow up *B. cinerea* development, three inoculated leaflets per genotype were collected 24 h post inoculation (hpi). Leaflets were visualized, after staining the pathogen with trypan blue as described by Fernández-Bautista et al. (2016), using a stereomicroscope (Olympus SZX9/Highlight 3,100). The area of stained hyphae and spores was measured with ImageJ by converting the pictures to a binary version (Fahrentrapp et al., 2019). Each treatment was performed with one leaflet and three replicates. Wilcox test was used to evaluate statistically significant differences between the genotypes.

### Quantification of *Botrytis cinerea* DNA on *Fragaria vesca* Leaves

For detection and quantification of *B. cinerea*, 3 weeks old strawberry leaflets were inoculated with three droplets of 5 μl of 10^6^ spores/mL *B. cinerea* and samples were collected by punching 1 cm circle discs around the infection site at 0, 6, 24, 48, 72 and 96 hpi. DNA was extracted according to the cetyltrimethylammonium bromide (CTAB) method (Tamari et al., 2013) with minor modifications (Supplementary Text 1). Each treatment was performed with two leaflets (six discs) and three replicates. The leaf samples were frozen immediately in liquid nitrogen and stored at −80°C.

Using genomic DNA of *B. cinerea* as template, quantitative polymerase chain reaction (qPCR) was performed with primers targeting the intergenic spacer (IGS) to specifically detect and quantify *B. cinerea* (Suarez et al., 2005). The qPCR reaction was performed in 20 μl total volume containing 2 μl of genomic DNA (gDNA) template, 2 μl of 5 μM forward and reverse primers (IGS), 4 μl of RNase-free-Milli-Q water and 10 μl of SYBR green (PowerUp^™^ SYBR^™^ Green Master Mix, Fisher Scientific, BIO-RAD) according to the manufacturer’s instruction. DNA from mycelium was used to generate calibration curves to quantify the amount of fungal DNA and Wilcox test was used to evaluate statistically significant differences between the genotypes. The sequences of the IGS primers can be found in Supplementary Table 1.

### Metabolite and Enzyme Activity Measurements

For the AsA, DHA, and the activity of CAT, APX, GPX and GR measurements, 3 weeks old leaves were used. For AsA and DHA, each treatment was performed with three leaflets and seven replicates. For the activity of CAT, APX, GPX and GR, each analysis was performed with three leaflets and three replicates. For H_2_O_2_, total phenolics, total flavonoids and primary metabolites measurements, trifoliate strawberry leaves were collected and the leaflets were analyzed. First, we showed that the three leaflets had similar resistance level to *B. cinerea* (Supplementary Figure 1). Then the left leaflets were used to evaluate their resistance against *B. cinerea* by the method described before (leaf inoculations and disease assays), while the middle and right leaflets were frozen immediately in liquid nitrogen and stored at −80°C for metabolite measurements. The samples were ground into a fine powder in liquid nitrogen before being measured.

#### Measurement of H_2_O_2_ Levels

H_2_O_2_ levels were measured as described by Junglee (Junglee et al., 2014) with minor modifications. Homogenized leaf material (50 mg) was extracted in 1 ml of solution containing 0.5 ml of 1 M KI, 0.25 ml of 1% trichloroacetic acid (TCA), and 0.25 ml of 10 mM potassium phosphate buffer (pH = 5.8) for 15 min at 4°C, centrifuged at 12,000 × *g* for 15 min at 4°C. Next, 200 μl of supernatant was placed in UV-microplate wells and incubated at room temperature for 20 min before measuring the absorbance at 350 nm *via* a spectrophotometer (SpectraMax^®^ Plus). The measurement was performed using three technical replicates and 18 biological replicates. Based on a standard curve, the H_2_O_2_ concentration was calculated as μmol/gFW. For tissue coloration background, a control was created using water instead of KI. *T*-test was used to evaluate statistically significant differences between the genotypes.

#### Measurement of Antioxidant Enzyme Activities

The extraction of CAT, APX, and GPX enzymes was performed as previously described (Van Rensburg et al., 2021) with minor modifications. Three weeks old strawberry trifoliate leaves were powdered in liquid nitrogen and 200 mg was extracted with 600 μl extraction buffer (100 mM phosphate buffer (pH 7.0), 0.1% Triton X-100, 15% glycerol, 1 mM phenylmethylsulfonyl fluoride (PMSF), 1 mM ascorbic acid, and 0.35 mM *β*-mercaptoethanol) by grinding with plastic micro pestles inside 2 ml Eppendorf tubes for 60 s and then incubated on ice for 1 h. Next, samples were centrifuged at 4°C for 10 min at 15,000 *× g*. Finally, the supernatants were kept at −80°C until further analysis, except for APX, which was measured immediately.

APX activity was measured as described previously (Van Rensburg et al., 2021). Reactions were carried out in 200 μl solution containing 185 μl of 100 mM phosphate buffer (pH 7.0),10 μl0.5 mM ascorbic acid and 5 μl enzyme extract. The absorbance of the reaction at 290 nm was measured using a spectrophotometer (SpectraMax^®^ Plus) at 10 s intervals for 5 min.

CAT activity was measured as previously described with minor modifications (Van Rensburg et al., 2021). The enzyme extract was diluted 10 times before being measured in a quartz cuvette with 1 ml 100 mM phosphate buffer (pH 7.0) and 10 μl diluted enzyme extract. Before starting the reaction, the background was monitored for 30 s. The decrease in absorbance at 240 nm was measured using a spectrophotometer (SpectraMax^®^ Plus) at 10 s intervals for 5 min. GR activity was measured as previously described with minor modifications (El-Shabrawi et al., 2010). The reagents used were 200 μl potassium phosphate buffer (100 mM, pH 7.6), 10 μl 1 mM ethylenediaminetetraacetic acid (EDTA), 10 μl 6 mM 5,5′-dithio-bis-(2-nitrobenzoic acid; DTNB), 20 μl 0.2 mM oxidized glutathione (GSSG) and 15 μl enzyme extract. Reaction was initiated by adding 10 μL 5 mM of NADPH. The increase in absorbance at 412 nm was measured using a spectrophotometer (SpectraMax^®^ Plus) at 10 s intervals for 5 min.

The activity of these three enzymatic reactions was determined using the reaction’s linear range, with enzyme activity reported in units U/mg protein, where 1 U is equal to the change in OD of 0.01 per min. The protein was measured using Coomassie Brilliant Blue G-250 (Sedmak and Grossberg, 1977).

The extraction of GPX was performed as described previously (Van Rensburg et al., 2021). After the extraction, the GPX activity was measured by the “glutathione peroxidase assay kit” according to the manufacturer’s protocol (Abbexa).

For the activity of CAT, APX, GPX and GR, *T*-test was used to evaluate statistically significant differences between the genotypes.

#### Measurement of Ascorbic Acid and Dehydroascorbate

Total AsA, AsA and oxidized dehydroascorbate (DHA) levels (total AsA-AsA) were measured as described by Stevens with minor modifications (Stevens et al., 2006). Briefly, homogenized leaf material (500 mg) was extracted in 1.3 ml of pre-cooled 6% TCA, incubated on ice for 15 min and centrifuged at 20,000 × *g* for 20 min at 4°C. Next, 90 μl of supernatant was used for measuring the absorbance at 550 nm using a spectrophotometer (SpectraMax^®^ Plus). The concentration of AsA and DHA was expressed in μg/g FW. *T*-test was used to evaluate statistically significant differences between the genotypes.

#### Measurement of Total Phenolics and Flavonoids

Total phenolic and flavonoid levels were determined following the method of Meng (Meng et al., 2019) with minor modifications. In short, homogenized leaf material (50 mg) was mixed with 1 ml of 80% methanol and sonicated for 20 min for 2 times with a 10 min stop in between. After centrifugation, the supernatant was collected for the measurement.

To determine total phenolics, 200 μl of supernatant was mixed thoroughly with 50 μl of the Folin–Ciocalteu reagent (BIPP Merck Life Science) for 3 min, followed by adding 50 μl of 10% Na_2_CO_3_ solution. Next, 700 μl of distilled water was added, the samples were left at room temperature for 2 h in dark, and then 200 μl of reaction solution was used to measure the absorbance at 765 nm. The total phenolic content was calculated as mg/g FW based on a standard curve established with gallic acid equivalent. To determine total flavonoids, 200 μl of supernatant was mixed with 60 μl of 5% NaNO_2_ in 800 μl distilled water for 5 min, then 120 μl of 10% AI(NO_3_)_3_ solution was added to react for 6 min. Finally, 400 μl of 1 M NaOH and 420 μl of distilled water was added and 200 μl of reaction solution from each sample was used to measure the absorbance at 510 nm. The total flavonoid content was calculated as mg/g FW based on a standard curve established with rutin equivalent (RE). Both measurements were performed using three technical replicates and 27 biological replicates for K1 and 44 biological replicates for T3. *T*-test was used to evaluate statistically significant differences between the genotypes.

#### Gas Chromatography–Mass Spectrometry Analysis

The metabolites were extracted from the leaf samples and derivatized using a previously described method (Hu et al., 2019) with minor modification. The homogenized samples (50 ± 2 mg) were extracted with 1.8 ml of pre-cooled methanol:water (4:1, v/v) by vortexing for 1 min and then sonicated at room temperature for 20 min. After centrifugation, 600 μL of the supernatant was transferred into two 2 ml Eppendorf tubes to measure sugars and acids separately.

For derivatization of sugars, shikimic acid and quininic acid, the same method as described by Terzoudis (Terzoudis et al., 2022) was used. For acids analysis, only 20 μl of 0.1 g/l 3-(4-hydroxyphenyl)-propionic acid was added to the samples as the internal standard and after drying, the derivatization was performed by adding 120 μL of methoxylamin-hydrochloride (20 g/l in pyridine, Sigma Aldrich) for 90 min at 37°C, 700 rpm. Next, 120 μl of BSTFA (Sigma Aldrich) was added and then incubated for 30 min at 60°C, 700 rpm. After centrifugation, 100 μL of supernatant was used for detection. In total, 18 detached leaves from each genotype were evaluated.

For separation and detection of analytes, GCMS was used in the same way as described by Terzoudis (Terzoudis et al., 2022). The concentration of primary metabolites was determined based on calibration curves and was calculated as mg/g FW. These results were first tested for normal distribution by the Shapiro–Wilk test, and then Wilcox and t-tests were used to assess statistically significant differences between the genotypes.

### qRT-PCR for Expression Analysis

Three weeks old leaves were collected to detect the expression of defense-related, ROS-related and specialized metabolism biosynthesis genes. Each analysis was performed with three trifoliate leaves and three biological replicates. Generalized linear models (glm) were used to evaluate statistically significant differences between the genotypes for defense related gene expression. Wilcox test was used to evaluate statistically significant differences between the genotypes for ROS-related and specialized metabolism biosynthesis-related gene expression. The leaf samples were frozen immediately in liquid nitrogen and stored at −80°C.

Total RNA was extracted using the CTAB method (Yu et al., 2012) combined with the RNeasy plant mini kit (Qiagen, Germany) with minor modifications (Supplementary Text 2). DNase treatment and cDNA synthesis were performed using DNAse I (New England Biolabs^®^ Inc) and SuperScript IV Reverse Transcriptase kit (ThermoFisher Scientific), respectively, according to the manufacturer’s instructions. Primers used for defense-related marker genes are shown in Supplementary Table 1. Quantitative Reverse Transcription PCR (qRT-PCR) reactions were performed in 20 μl containing 6 μl of cDNA template, 2 μl of 5 μM forward and reverse primers, and 10 μl of SYBR green. The parameters for performing qRT-PCR are similar to the qPCR described earlier, except for the number of amplification cycles (40 instead of 35). The relative quantification of gene products was based on the ∆Ct method (Schmittgen and Livak, 2008). Transcript levels were normalized to the reference genes *HISTONE H4* (*H4*) and *UBIQUITIN-PROTEIN LIGASE* (*UBC9*; Zhang et al., 2018; Jose et al., 2020). All primers can be found in Supplementary Table 1.

### Data Analysis

For GC–MS, the data was analyzed by means of Principal Component Analysis (PCA) and Partial Least Squares Regression (PLSR) both on the correlation matrices using The Unscrambler^®^ X (10.5.1) according to Hu (Hu et al., 2019).

For the other experiments, statistical analysis was conducted using R.^1^ Different statistical tests were used for the different datasets and indicated in previous method and the figure legends. For the boxplots, the box limits are the 25th and 75th percentile, the middle line in the individual boxes represents the median, the white point in the individual boxes marks the mean, whiskers extend to 1.5-fold the interquartile range of the 25th and 75th percentiles. For bar plots, values are represented as means of replicates with standard errors shown by vertical bars.

## Results

### Genotype K1 Is More Resistant to *Botrytis cinerea* Than T3

**Figure 1:**
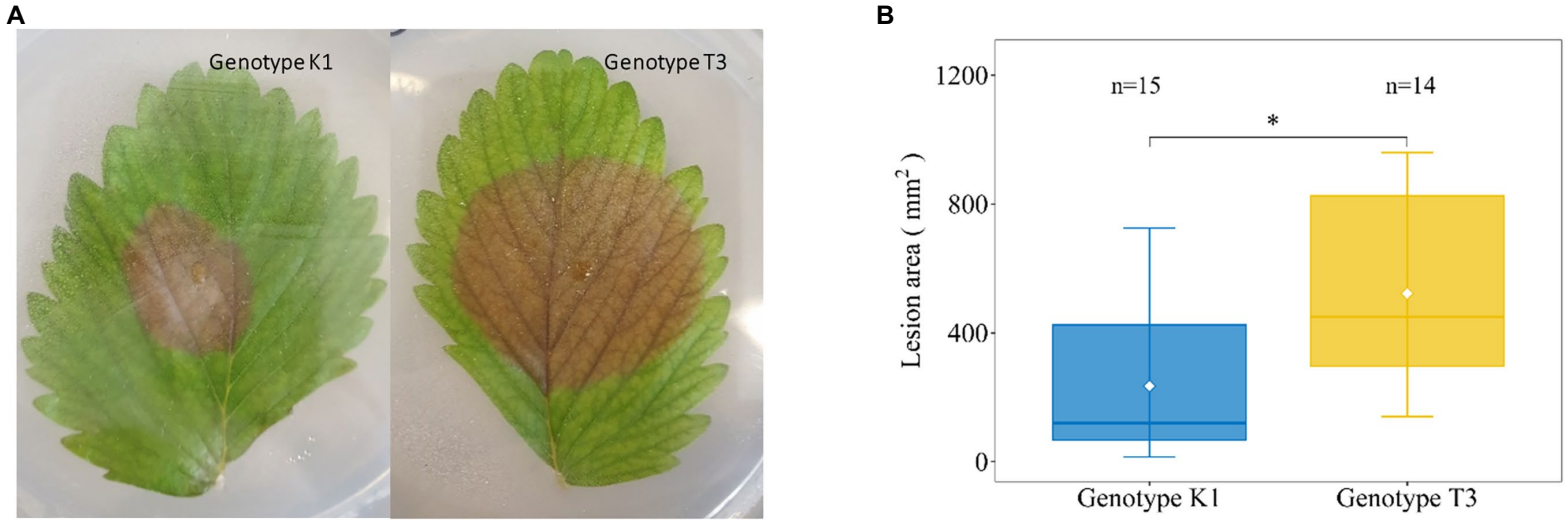
Representative picture of disease symptoms **(A)** and lesion area **(B)** on strawberry leaves of Kreuzkogel 1 (K1) and Tenno 3 (T3) at 5 d post *Botrytis cinerea* inoculation. The asterisk ‘*’ indicates significant differences among two genotypes by Wilcox test at *p* < 0.05.

In an initial screen for altered leaf susceptibility of different *F. vesca* genotypes against *Botrytis cinerea* (Supplementary Figure 2), we identified two genotypes for further research, i.e., *Fragaria vesca* ssp. *vesca* Kreuzkogel 1 (K1) and *F. vesca* ssp. *vesca* Tenno 3 (T3). We confirmed in an independent experiment that at 5 dpi, *B. cinerea* inoculation results in significant larger lesions in T3 compared to K1 (624 mm^2^ vs. 227 mm^2^; Figure 1). Based on the DSI for K1 (42%) and T3 (69%), we defined the genotypes as moderately resistant and susceptible, respectively.

**Figure 2:**
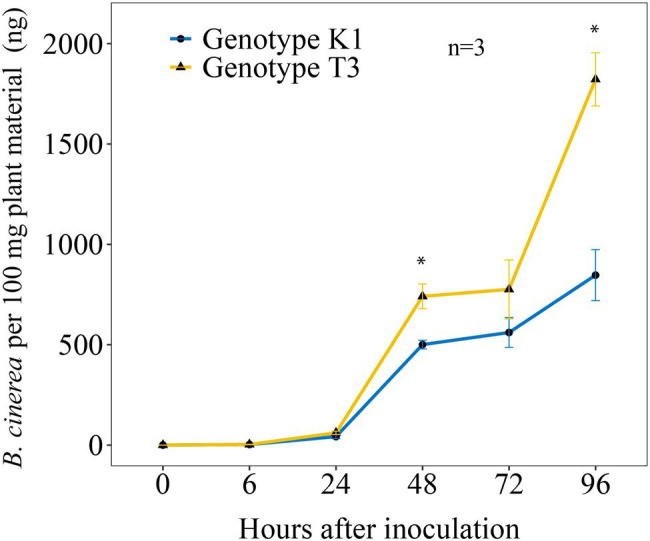
Amount of *B. cinerea* B05.10 DNA per 100 mg leaves at different time points post inoculation (0, 6, 24, 48, 72 and 96 h post *B. cinerea* inoculation) in Kreuzkogel 1 (K1) and Tenno 3 (T3). The asterisk ‘*’ indicates significant differences among two genotypes by Wilcox test at *p* < 0.05. Values are represented as means of three replicates with standard error shown by vertical bars.

qPCR was performed to analyze the amount of *B. cinerea* DNA in both strawberry genotypes at different time points post inoculation (Figure 2). A significantly higher amount of *B. cinerea* DNA of 48 and 115.2% was observed at 48 and 96 hpi, respectively, in genotype T3 compared to K1.

**Figure 3:**
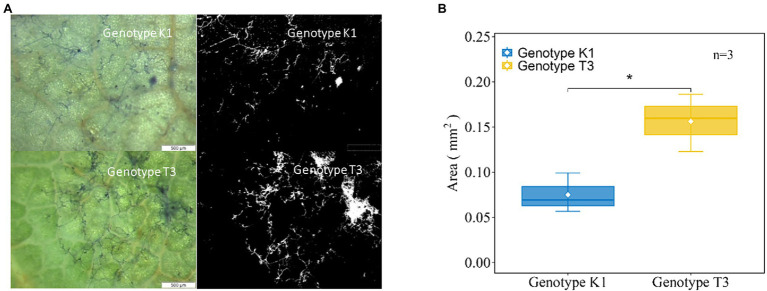
**(A)** Microscopic (left) and binary image (right) of the hyphal growth at 24 h post inoculation (hpi) for Kreuzkogel 1 (K1) and Tenno 3 (T3). The microscopic evaluation was done by trypan blue staining of fungal growth and the binary image was converted and analyzed by the ImageJ software. **(B)** Total area (mm^2^) of hyphae on leaves of K1 and T3 at 24 hpi. The asterisk ‘*’ indicates significant difference among two genotypes by Wilcox test at *p* < 0.05.

Next, fungal growth was microscopically observed at 24 hpi (Figure 3). More hyphal growth was observed in T3 compared to K1 (Figure 3A). The hyphal growth area was calculated using ImageJ software confirming that hyphal growth on T3 leaves was significantly higher (2.08-fold) compared to K1 at 24 hpi (Figure 3B). Altogether, these data show that K1 is more resistant against *B. cinerea* than T3.

### Induced Defense Responses Have a Minor Role in *Botrytis cinerea* Resistance for Both Genotypes

**Figure 4:**
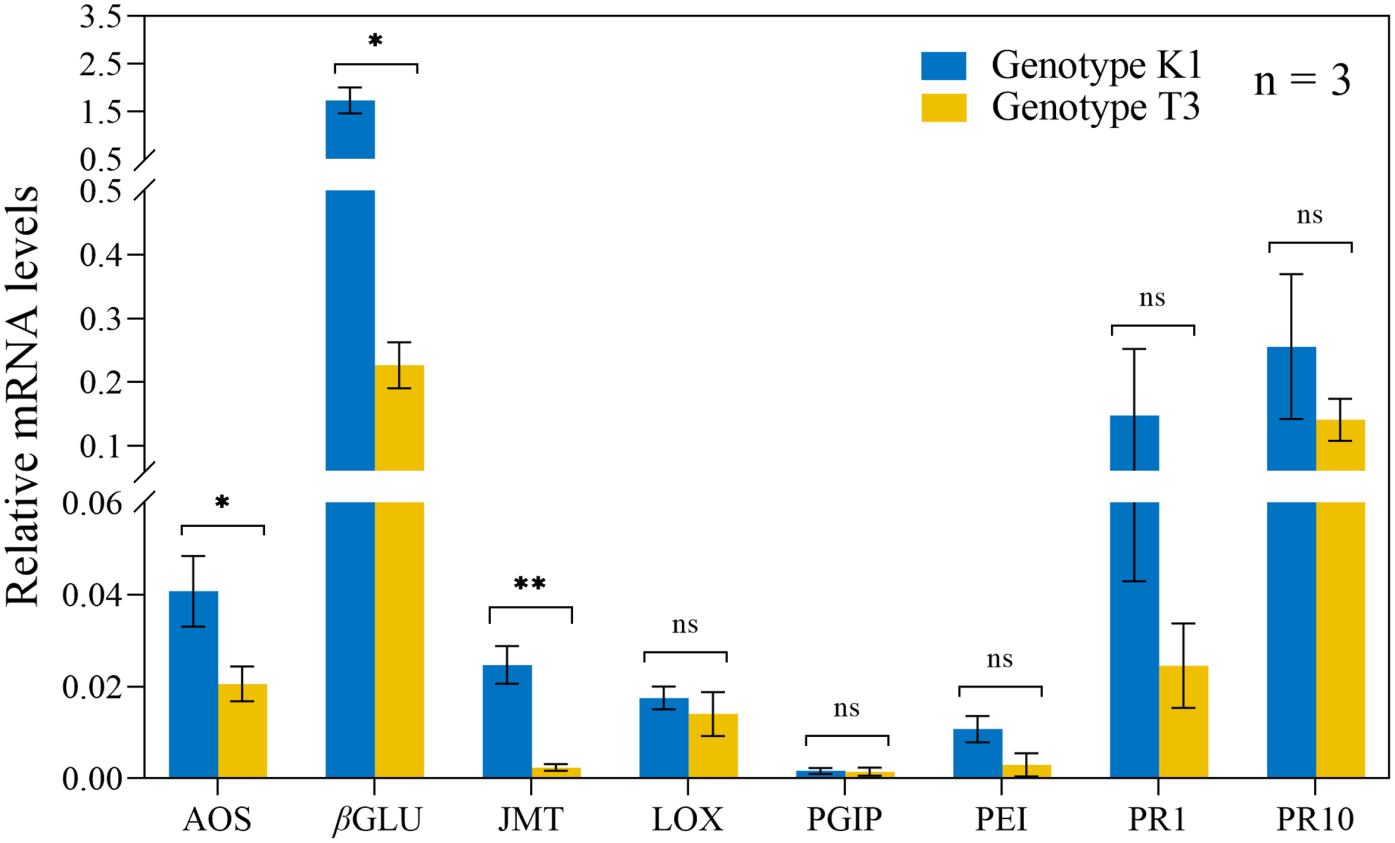
Relative expression levels of eight defense-related genes in leaves of Kreuzkogel 1 (K1) and Tenno 3 (T3), prior to *B. cinerea* inoculation, were measured *via* qRT-PCR. All values were normalized to the expression level of the *H4* and *UBC9* housekeeping genes. Significant differences between two genotypes, based on the generalized linear models (glm), are represented by ns *p* > 0.05, ^*^p < 0.05, ^**^p < 0.01. *AOS, ALLENE OXIDE SYNTHASE; βGLU, β-1,3-GLUCANASE; JMT, JASMONIC ACID CARBOXYL METHYLTRANSFERASE; LOX, LIPOXYGENASE; PGIP, POLYGALACTURONASE INHIBITOR PROTEIN; PEI, PECTINESTERASE INHIBITOR; PR1, PATHOGENESIS-RELATED PROTEIN FAMILY 1; PR10, PATHOGENESIS-RELATED PROTEIN FAMILY 10.*

To evaluate the expression of *AOS, PAL, JASMONIC ACID CARBOXYL METHYLTRANSFERASE* (*JMT*)*, LOX, PGIP, PECTINESTERASE INHIBITOR* (*PEI*)*, PATHOGENESIS RELATED PROTEIN FAMILY 1* (*PR1*) and *PR10* upon *B. cinerea* inoculation, qRT-PCR analysis on both genotypes was performed at 24 and 48 hpi (Supplementary Figure 3). No clear indication for a stronger defense response was found for both genotypes. However, before *B. cinerea* inoculation, a significantly higher expression of *AOS, βGLU* and *JMT* of 1.98-fold, 10.46-fold and 7.65-fold, respectively, was detected in K1 compared to T3 (Figure 4). This result suggests that the increased resistance of K1 against *B. cinerea*, compared to T3 is due to the basal resistance mechanisms rather than induced defense responses.

### Lower Levels of H_2_O_2_ in K1 Correlate With Higher Resistance Against *Botrytis cinerea*

**Figure 5:**
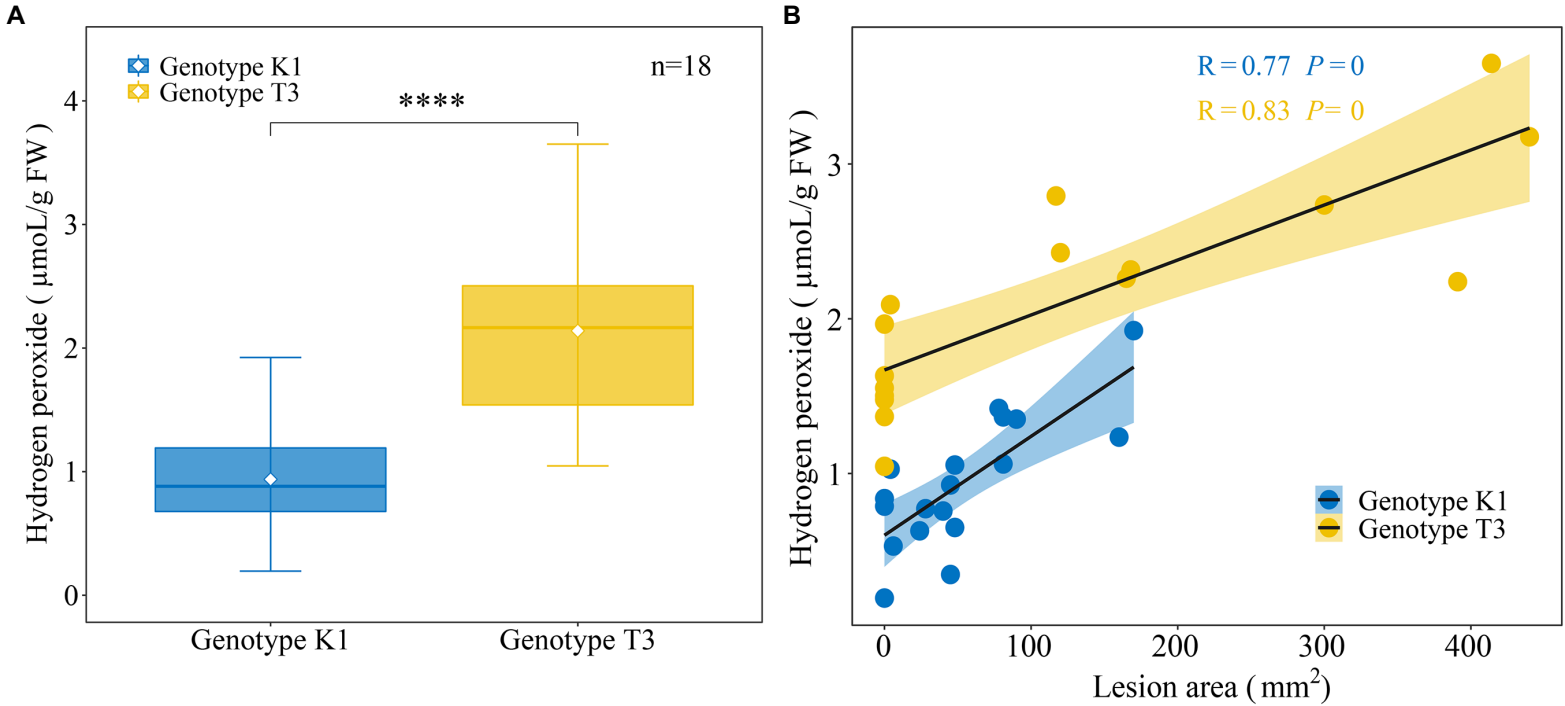
Analysis of H_2_O_2_ production of leaves of Kreuzkogel 1 (K1) and Tenno 3 (T3). **(A)** The amount of H_2_O_2_ in strawberry middle and right leaflets for K1 and T3 without *B. cinerea* inoculation. The asterisk ‘*’ indicates significant differences among two genotypes by *t*-test at *p* < 0.0001. **(B)** The correlation between lesion area, measured on strawberry left leaflets at 5 dpi H_2_O_2_ in strawberry middle and right leaflets for K1 and T3, respectively, without *B. cinerea* inoculation.

It has been previously reported that in one to five week old leaves of *F. × ananassa* strawberry plants, H_2_O_2_ levels correlated positively with gray mold disease severity (Meng et al., 2019). Therefore, we aimed at investigating the correlation between constitutive H_2_O_2_ levels in strawberry leaves and *B. cinerea* resistance for both genotypes. We first determined the amount of H_2_O_2_ in the two strawberry genotypes (Figure 5). The amount of H_2_O_2_ (μmoL/g FW) was significantly lower in K1 (0.93 ± 0.10) compared to T3 (2.14 ± 0.18; Figure 5A). Next, the H_2_O_2_ levels were correlated with *B. cinerea* resistance for both genotypes. Lesion area (mm^2^) showed a high positive correlation with H_2_O_2_ levels for K1 (*R* = 0.77, *p* = 0) and T3 (*R* = 0.83, *p* = 0). Accordingly, T3 leaves showed a larger lesion area and higher H_2_O_2_ content compared to K1 (Figure 5B), suggesting that lower levels of H_2_O_2_ are important for the increased resistance against *B. cinerea*, which is in line with previous observations (Govrin and Levine, 2000; Khanam et al., 2005; Meng et al., 2019).

### Higher Expression Levels and Activity of ROS Scavenging Enzymes Were Observed in K1

The level of H_2_O_2_ is regulated by the expression and activity of ROS production and ROS scavenging enzymes (Foyer and Noctor, 2016). Here, we analyzed the enzymatic and non-enzymatic systems involved in controlling H_2_O_2_ levels to investigate the mechanisms underlying the difference in H_2_O_2_ levels between the two genotypes.

**Figure 6:**
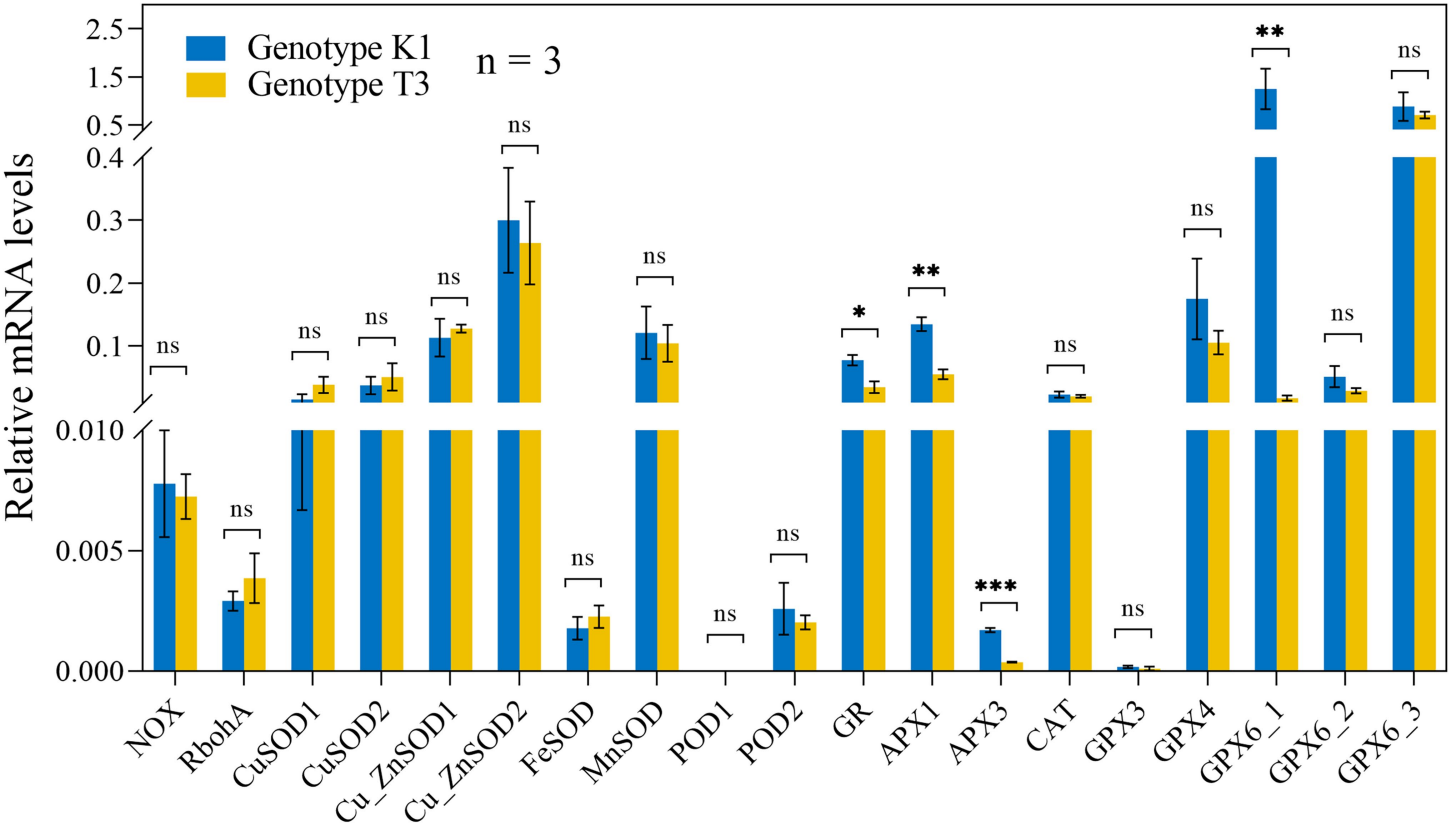
Relative expression levels of genes involved in H_2_O_2_ production and scavenging in leaves of Kreuzkogel 1 (K1) and Tenno 3 (T3) without *B. cinerea* inoculation were measured *via* qRT-PCR. All values were normalized to the expression level of the *H4* and *UBC9* housekeeping genes. Significant differences between two genotypes, based on the Wilcox test, are represented by ns *p* > 0.05, ^*^p < 0.05, ^**^p < 0.01 and ^***^p < 0.001. *NOX, NADPH OXIDASE; RBOHA, RESPIRATORY BURST OXIDASE HOMOLOGS; SOD, SUPEROXIDE DISMUTASE; CAT, CATALASE; POD, PEROXIDASE; GR, GLUTATHIONE REDUCTASE; APX: ASCORBATE PEROXIDASE, GPX, GLUTATHIONE PEROXIDASE*.

**Figure 7:**
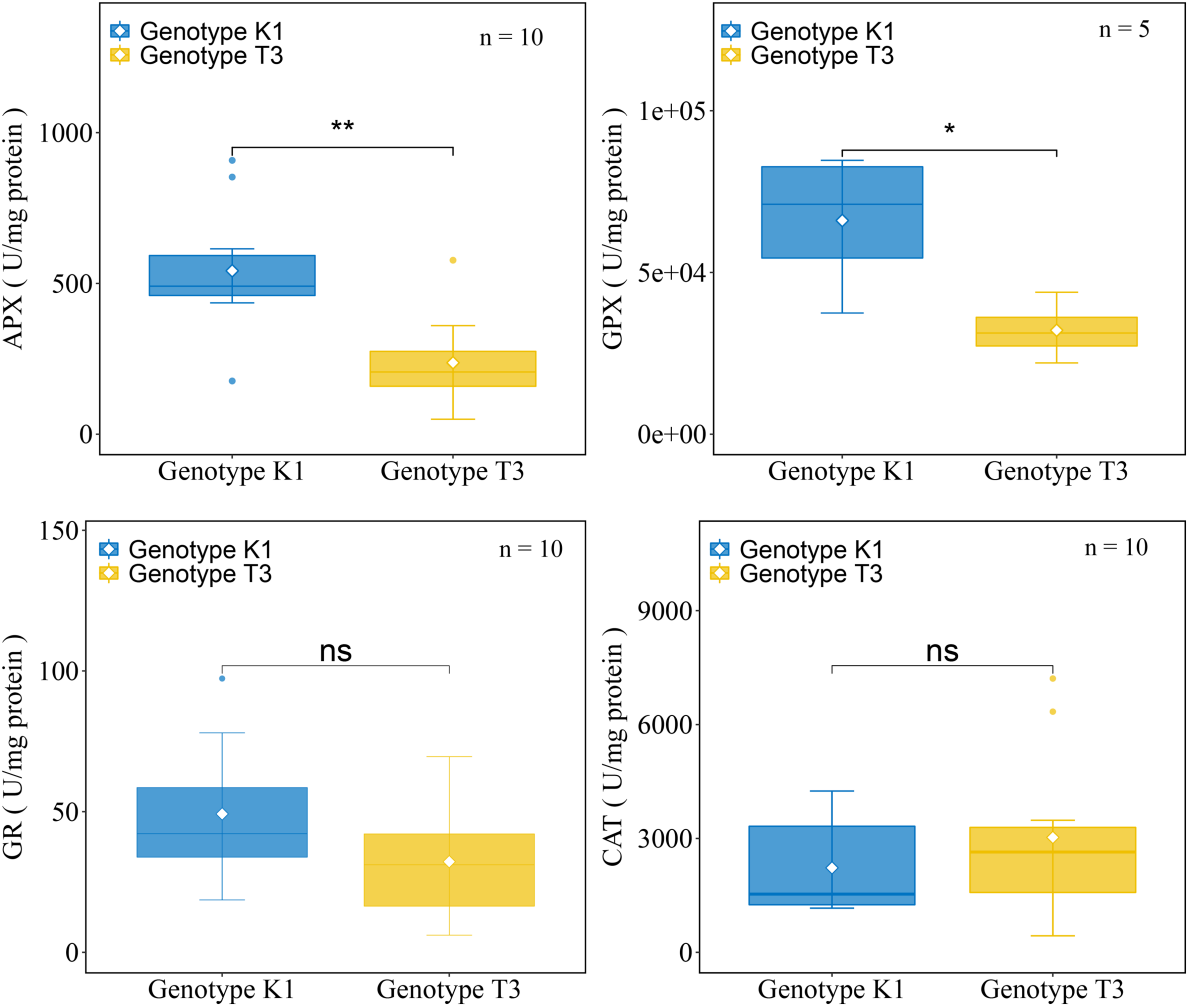
The activity of ROS scavenging enzymes in leaves of Kreuzkogel 1 (K1) and Tenno 3 (T3) without *B. cinerea* inoculation. Significant differences between two genotypes, based on a *t*-test, are represented by ns *p* > 0.05, ^*^p < 0.05 and ^**^p < 0.01. The individual points represent the outliers. APX, ascorbate peroxidase; GPX, glutathione peroxidase; GR, glutathione reductase; CAT, catalase.

No significant differences in the transcript levels of *NADPH OXIDASE* (*NOX*)*, RESPIRATORY BURST OXIDASE HOMOLOGS* (*RBOHA*), and *SUPEROXIDE DISMUTASE* (*SOD*) isoforms were observed between the genotypes (Figure 6). Also, the expression of *CATALASE* (*CAT*) and *PEROXIDASE* (*POD*), which catalyze the conversion of H_2_O_2_ to H_2_O and O_2_ did not differ significantly between the two genotypes (Figure 6). Glutathione reductase (GR) and ascorbate peroxidase (APX) as well as glutathione peroxidase (GPX) are important enzymes of the AsA-GSH cycle and GPX cycle, respectively, and the gene expression of different isoforms was tested in both genotypes. Transcript levels of *GR*, two isoforms of *APX* gene (*APX1* and *APX3*) and one of the five *GPX* genes (*GPX6_1*) were significantly higher expressed in K1 compared to T3 (Figure 6). In addition, the activity of the antioxidant enzymes GPX, APX, GR and CAT were analyzed in both genotypes. GPX and APX activities were significantly higher in K1 compared to T3, while CAT activity did not differ between two genotypes (Figure 7) and these results correlate nicely with their corresponding gene expression. GR activity was not different between the two genotypes (Figure 7). Overall, we can propose that the lower H_2_O_2_ in K1 compared to T3 is due to the higher expression level and activity of ROS scavenging enzymes.

### Higher Levels of Ascorbic Acid and Phenolic Compounds Were Observed in K1

**Figure 8:**
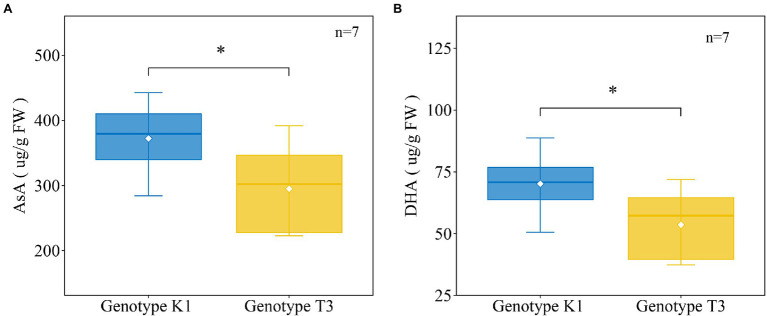
The amount of ascorbic acid (AsA) **(A)**, dehydroascorbate (DHA) **(B)** in strawberry leaves of Kreuzkogel 1 (K1) and Tenno 3 (T3) without *B. cinerea* inoculation. The asterisk ‘*’ indicates significant differences among the two genotypes determined by a *t*-test at *p* < 0.05.

In addition to antioxidant enzymes, there are also non-enzymatic systems to scavenge H_2_O_2_ in plants, among which AsA has an important H_2_O_2_ scavenging function in the plant cell (Takahama and Oniki, 1997). In addition, APX, which metabolizes H_2_O_2_ to H_2_O using AsA as a specific electron donor, showed significant differences in gene expression and enzyme activity between the two genotypes (Figures 6, 7). Therefore, the levels of the major antioxidant AsA and its oxidized form dehydroascorbic acid (DHA) were measured. The amount of AsA was significantly higher in K1 (302.07 ± 43.62 μg/g FW) compared to T3 (241.40 ± 56.45 μg/g FW; Figure 8A). Similarly, the amount of DHA was significantly higher in K1 (70.18 ± 12.56 μg/g FW) compared to T3 (53.58 ± 14.94 μg/g FW; Figure 8B).

**Figure 9:**
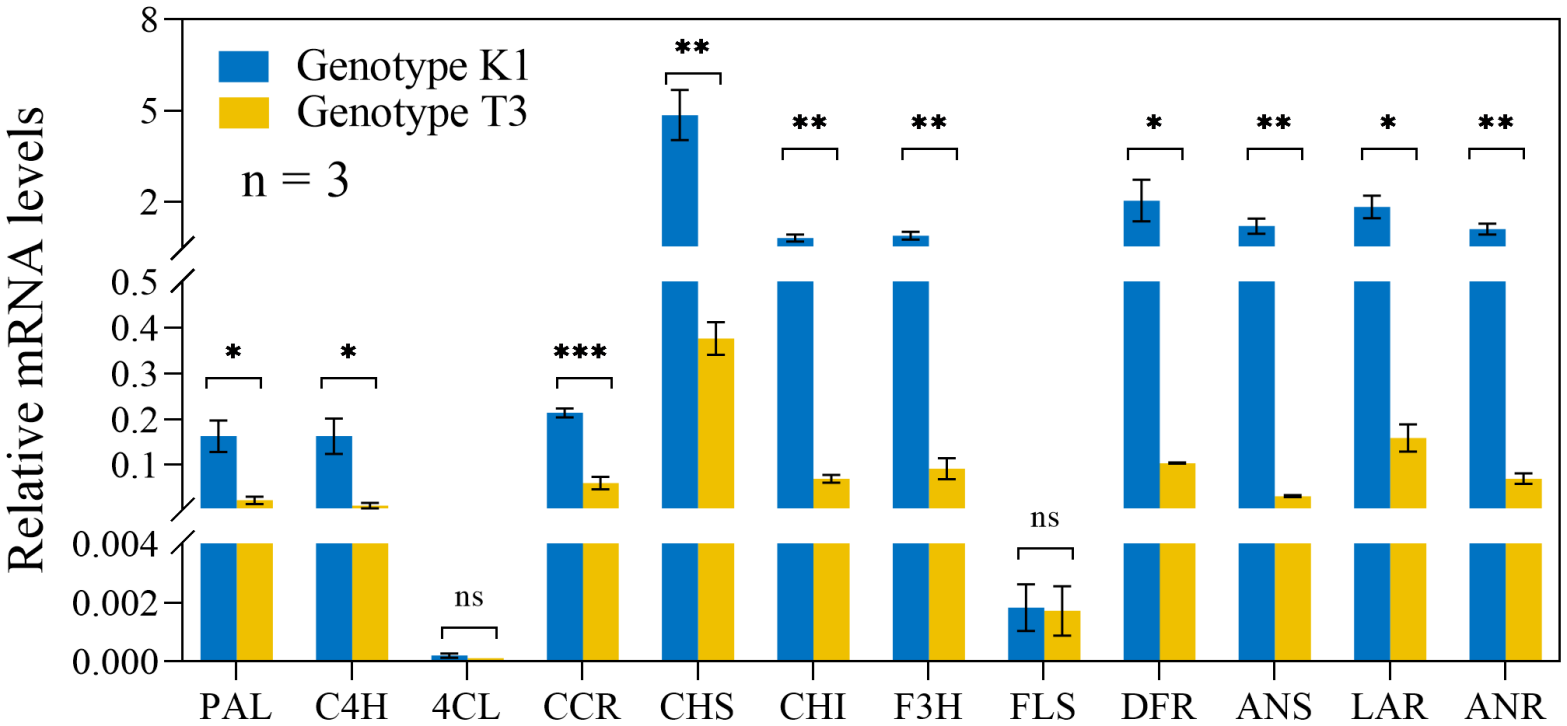
Relative expression levels of genes involved in the phenylpropanoid pathway in leaves of Kreuzkogel 1 (K1) and Tenno 3 (T3) without *B. cinerea* inoculation were measured *via* qRT-PCR. All values were normalized to the expression level of the *H4* and *UBC9* housekeeping genes. Significant differences between two genotypes, based on the Wilcox test, are represented by ns *p* > 0.05, ^*^p < 0.05, ^**^p < 0.01 and ^***^p < 0.001. *PAL, PHENYLALANINE AMMONIA LYASE; C4H, CINNAMIC ACID 4-HYDROXYLASE; 4CL, 4-COUMAROYL-COA LIGASE; CCR: CINNAMOYL-COA REDUCTASE; CHS, CHALCONE SYNTHASE; CHI, CHALCONE ISOMERASE; F3H, FLAVANONE 3-HYDROXYLASE; FLS, FLAVONOL SYNTHASE; DFR, DIHYDROFLAVONOL REDUCTASE; ANS, ANTHOCYANIDIN SYNTHASE; LAR, LEUCOANTHOCYANIDIN REDUCTASE; ANR, ANTHOCYANIDIN REDUCTASE*.

**Figure 10:**
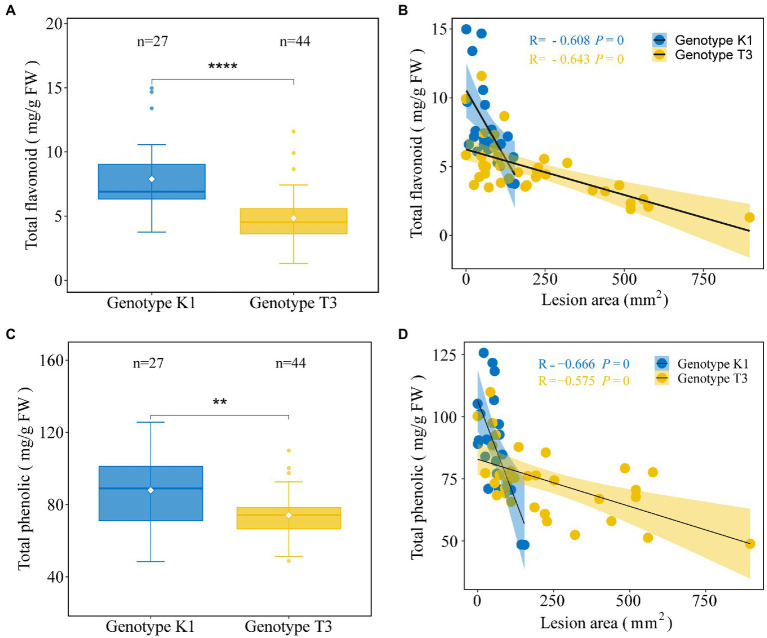
Analysis of total flavonoid and total phenolic contents of leaves of Kreuzkogel 1 (K1) and Tenno 3 (T3). The amount of total flavonoid **(A)** and total phenolic **(C)** in strawberry middle and right leaflets for K1 and T3 without *B. cinerea* inoculation. Significant differences between two genotypes, based on a *t*-test, are represented by ^**^p < 0.01 and ^****^p < 0.0001. The individual points represent the outliers. The correlation between lesion area, measured on strawberry left leaflets, at 5 days after *B. cinerea* inoculation and total flavonoid **(B)** and total phenolic contents **(D)** in strawberry middle and right leaflets without *B. cinerea* inoculation for K1 and T3, respectively.

Also phenolics can act as ROS scavengers and are important metabolites against pathogens (Pourreza, 2013). High amounts of flavonoids and phenolics have previously been shown to be associated with increased resistance against *B. cinerea* (Ray et al., 2015). Here, except for *4-COUMAROYL-COA LIGASE* (*4CL*) and *FLAVONOL SYNTHASE* (*FLS*), the expression of biosynthesis genes of the phenylpropanoid pathway such as *PHENYLALANINE AMMONIA LYASE* (*PAL*)*, CINNAMIC ACID 4-HYDROXYLASE* (*C4H*)*, CINNAMOYL-COA REDUCTASE* (*CCR*)*, CHALCONE SYNTHASE* (*CHS*)*, CHALCONE ISOMERASE* (*CHI*)*, FLAVANONE 3-HYDROXYLASE* (*F3H*)*, DIHYDROFLAVONOL REDUCTASE* (*DFR*)*, ANTHOCYANIDIN SYNTHASE* (*ANS*)*, LEUCOANTHOCYANIDIN REDUCTASE* (*LAR*) AND *ANTHOCYANIDINN REDUCTASE* (*ANR*) was significantly higher in K1 compared to T3 (Figure 9). Accordingly, also the total flavonoid and total phenolic contents were higher in K1 compared to T3 (Figures 10A,C). In addition, consistent with previous observations, total flavonoid and total phenolic contents of both genotypes correlated negatively with lesion area (Figures 10B,D; Ray et al., 2015).

### Several Primary Metabolites Contribute to the Higher Resistance of K1

**Figure 11:**
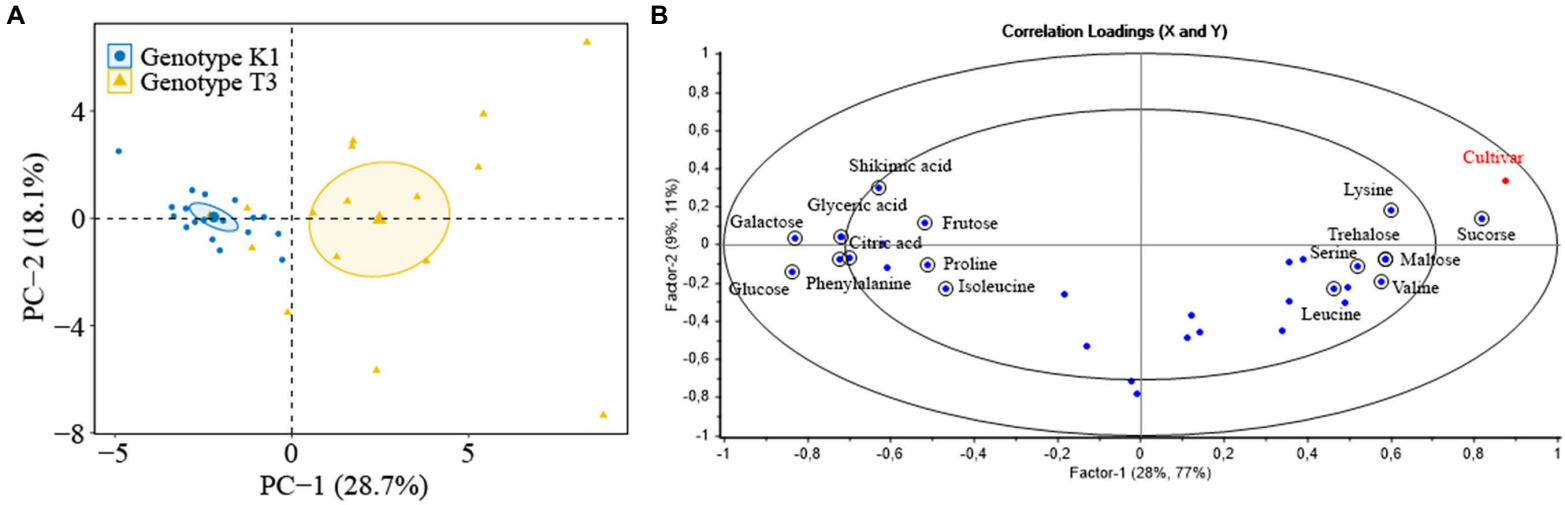
**(A)** PCA score plot of primary metabolites of strawberry leaves of Kreuzkogel 1 (K1) and Tenno 3 (T3). One dot represents one strawberry leaf, and PC-1 and PC-2 refer to the first and the second factor used to explain the variance. **(B)** Correlation loading plots of PLSR analysis with the primary metabolite profile of strawberry leaves of K1 and T3 as variables and cultivar (category) as response. The outer ellipse indicates 100% of explained variance, while the inner ellipse indicates 50% of explained variance. One blue dot represents one specific metabolite (X-factor), and encircled metabolites contributed to the separation of two genotypes which selected by jack-knifing test. The red dot represent the category cultivar (Y-factor).

Primary metabolites are precursors of specialized metabolites and several metabolites (sugars and amino acids) contribute to the physiological or morphological adaptations of strawberry plants to restrict pathogen invasion (Hu et al., 2019). Principal component analysis (PCA) and partial least squares regression (PLSR) were performed to investigate the effect of primary metabolites on plant resistance in leaves of T3 and K1. PCA analysis showed variance between the two genotypes (Figure 11A). Most variation (28.7%) was captured by PC 1 which was related to differences between the two genotypes, while PC 2 (18.1%) described the variation within the genotypes. In the PLSR correlation loading plot (Figure 11B), the metabolites, selected based on a jack-knifing test, that contributed most to the separation of the two genotypes were fructose, glucose, galactose, sucrose, trehalose, maltose, shikimic acid, citric acid, glyceric acid, phenylalanine, isoleucine, valine, proline, serine, lysine and leucine. The concentration of these metabolites and the correlation with lesion area for two genotypes are shown in Table 1.

**Table 1:** Important primary metabolites based on the PLSR contributed to the separation of genotype Kreuzkogel 1 (K1) and Tenno 3 (T3).

Metabolites	RT (min)	Genotype K1			Genotype T3		
		Content (mg/g FW)	*r* ^c^	*p* ^d^	Content (mg/g FW)	*r* ^c^	*p* ^d^
Fructose^a^	8.31	0.906 ± 0.132	−0.64	0	0.514 ± 0.120^****^	−0.28	0.29
Glucose^a^	8.52	0.651 ± 0.038	−0.57	0.01	0.287 ± 0.047^****^	−0.63	0.01
Galactose^a^	8.78	0.661 ± 0.068	−0.73	0	0.248 ± 0.045^****^	−0.58	0.02
Sucrose^a^	12.29	2.476 ± 0.050	0.86	0	4.031 ± 0.263^****^	0.81	0
Trehalose^a^	12.74	0.011 ± 0.001	−0.12	0.63	0.021 ± 0.003^***^	−0.25	0.36
Maltose^a^	12.87	0.195 ± 0.024	−0.12	0.63	0.370 ± 0.045^***^	−0.24	0.37
Shikimic acid^a^	8.01	0.236 ± 0.067	−0.41	0.09	0.049 ± 0.005^****^	−0.63	0.01
Citric acid^b^	33.51	1.252 ± 0.199	−0.86	0	0.387 ± 0.036^***^	−0.79	0
Glyceric acid^a^	24.11	0.145 ± 0.023	−0.04	0.87	0.029 ± 0.005^****^	−0.29	0.27
Phenylalanine^a^	26.23	0.007 ± 0.001	−0.78	0	0.004 ± 0.001^****^	−0.58	0.02
Isoleucine^b^	20.27	0.019 ± 0.002	−0.2	0.43	0.013 ± 0.001^**^	−0.16	0.55
Valine^b^	18.92	0.008 ± 0.001	−0.32	0.19	0.013 ± 0.001^***^	−0.34	0.2
Proline^a^	20.93	0.136 ± 0.041	0.07	0.79	0.010 ± 0.003^**^	−0.23	0.4
Serine^a^	24.70	0.009 ± 0.001	−0.36	0.14	0.051 ± 0.015^***^	−0.18	0.51
Lysine^a^	30.39	0.003 ± 0.000	0.81	0	0.005 ± 0.001^*^	0.89	0
Leucine^b^	19.76	0.179 ± 0.020	−0.12	0.63	0.292 ± 0.042^*^	0.11	0.68

Significant differences between two genotypes are represented,

* *p* < 0.05,

** *p* < 0.01,

*** *p* < 0.001,

**** *p* < 0.0001.

a Wilcox test to check the significant difference between two groups.

b *t* test to check the significant difference between two groups.

c Pearson’s correlation coefficient *r* between lesion area measured on strawberry left leaflets at 5 dpi and metabolites in strawberry middle and right leaflets for K1 and T3, respectively, without *B. cinerea* inoculation.

d Probabilities of significance of linear models relating lesion area measured on strawberry left leaflets at 5 dpi to metabolites in strawberry middle and right leaflets for K1 and T3, respectively, without *B. cinerea* inoculation.

As reported in Table 1, significant differences were observed for 16 metabolites selected from the PLSR approach between the two genotypes. In addition, three other metabolites that were not selected by PLSR analyses, fucose, quininic acid, and fumaric acid, also showed significant differences between the two genotypes (Supplementary Table 2). Furthermore, the amount of 11 metabolites is significant higher in K1 compared to T3 while for 9 metabolites the amount is lower in K1 compared to T3 (Table 1; Supplementary Table 2). Among the measured metabolites, glucose, galactose, citric acid and phenylalanine correlated negatively with LA, while sucrose and lysine correlated positively (*p* < 0.05). The amount of trehalose, maltose, glyceric acid, isoleucine, valine, proline, serine and leucine did not significantly correlate with lesion area in any of the two genotypes, while the amount of fructose and shikimic acid correlated with lesion area in genotype K1 and genotype T3, respectively.

## Discussion

Strawberry is an important fruit crop grown in more than 70 countries (Van Lammerts Bueren et al., 2011). Gray mold caused by *B. cinerea* leads to substantial economic losses worldwide (Williamson et al., 2007). *B. cinerea* can infect multiple parts of the strawberry plant including fruit, flowers and leaves (Petrasch et al., 2019). Currently, fungicides are used to control *B. cinerea* on strawberries, which has resulted in the increased development of fungicide-resistant strains (Leroch et al., 2011, 2013; Veloukas et al., 2011; Bestfleisch et al., 2013). To solve this problem, breeding for *B. cinerea*-resistant genotypes could be an alternative management strategy (Roudeillac, 2003). In this study, a moderately resistant and susceptible *F. vesca* genotype were analyzed for H_2_O_2_ production, ASA, primary and specialized metabolites production, antioxidant enzyme activities and the expression of defense-related genes to investigate the constitutive resistance mechanisms present in both genotypes.

### The Moderately Resistant Genotype K1 Limits *Botrytis cinerea* Growth Compared to the Susceptible Genotype T3

Our results on symptom development, qPCR and microscopy after *B. cinerea* inoculation demonstrate that K1 is more resistant to *B. cinerea* than T3 (Figures 1–3). A previous study, investigating the rate of adult *Drosophila suzukii* emergence on K1 and T3 fruit exposed to egg-laying fly females, found that less fly emergences occurred in K1 compared to T3 (Gong et al., 2016). Consequently, K1 may represent a valuable germplasm for breeding new varieties with resistance to biotic stress (Bestfleisch et al., 2015).

We found a significant higher development of hyphae on T3 leaves compared to K1 at 24 hpi (Figure 3), but for *B. cinerea* DNA, significant differences were observed only after 48 hpi (Figure 2). This suggests that the sensitivity of the qPCR assay is not high enough to distinguish the differences before 24 hpi. Overall, K1 can repress the *B. cinerea* hyphal growth at the early infection stage and possibly inhibit spore germination. A previous study on grapes reported a similar result where *B. cinerea* spores germinated at a lower rate on a resistant cultivar than on a susceptible cultivar (Wan et al., 2015). Several antioxidants can inhibit *B. cinerea* growth, for example, catechin and quercetin-3-galactoside inhibit germ tube elongation while gallic acid inhibits spore germination (Tao et al., 2010). In this case, antioxidants, including AsA, phenolics, flavonoids and primary metabolites, contributed to the growth restriction of *B. cinerea* in K1 compared to T3 (Figure 10; Table 1).

### Summary of Potential Mechanisms for Higher Resistance of K1 to *Botrytis cinerea* Than T3

**Figure 12:**
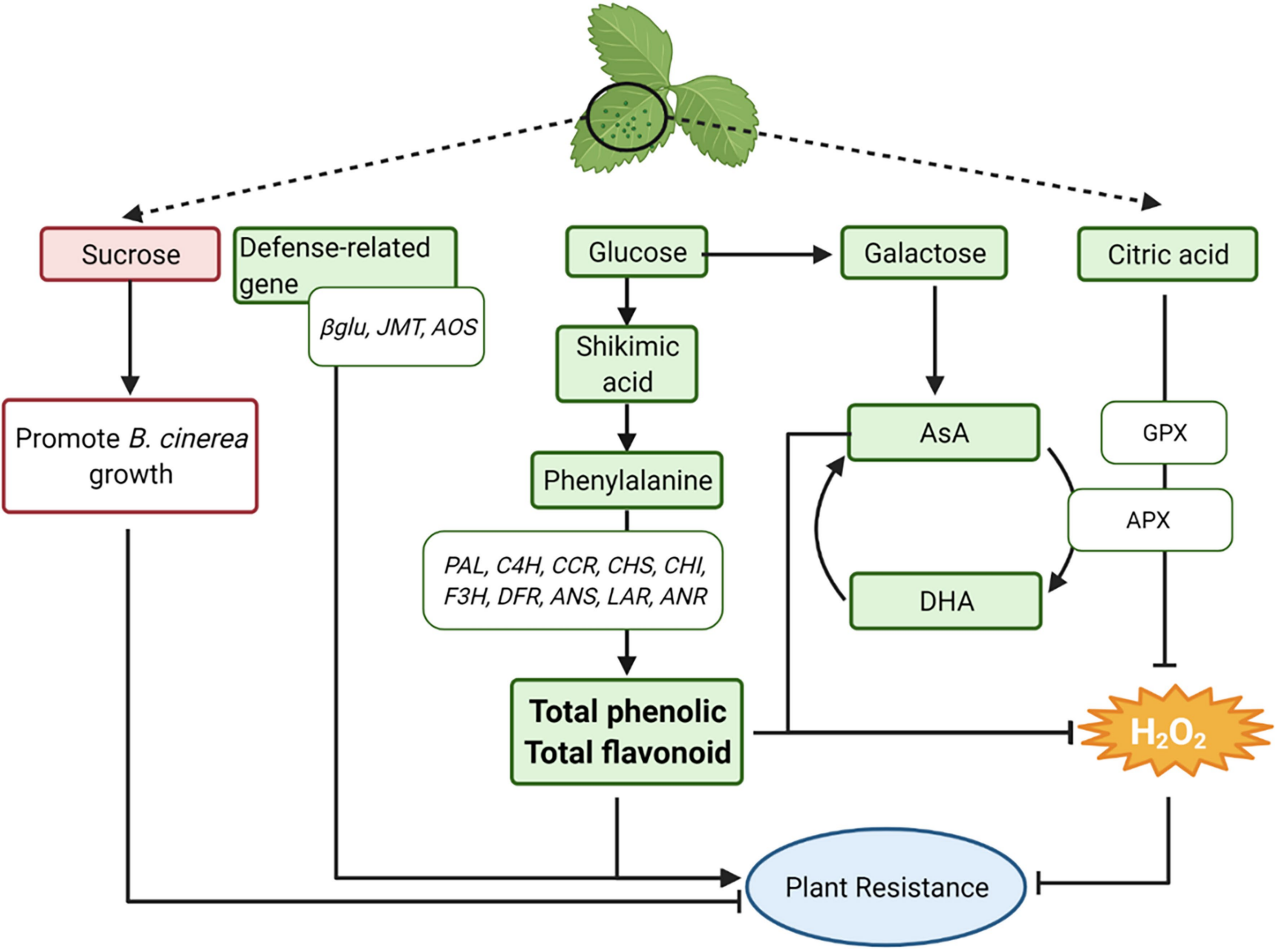
A conceptual model illustrating potential resistance mechanisms to *B. cinerea* in the more tolerant woodland strawberry *F. vesca* ssp. *vesca* Kreuzkogel 1 (K1) including primary metabolites, H_2_O_2_, phenolic compounds, ascorbic acid and defense-related genes. A detailed description of the figures can be found in the “Summary of Potential Mechanisms for Higher Resistance of K1 to *B. cinerea* Than T3”. The green boxes represent a higher content of the specific metabolites/genes in genotype K1 compared to T3, while the red boxes represent a lower content. *βGLU: β-1,3-GLUCANASE, JMT: JASMONIC ACID CARBOXYL METHYLTRANSFERASE, AOS: ALLENE OXIDE SYNTHASE; PAL, PHENYLALANINE AMMONIA LYASE; C4H, CINNAMIC ACID 4-HYDROXYLASE; 4CL, 4-COUMAROYL-COA LIGASE; CCR: CINNAMOYL-COA REDUCTASE; CHS, CHALCONE SYNTHASE; CHI, CHALCONE ISOMERASE; F3H, FLAVANONE 3-HYDROXYLASE; FLS, FLAVONOL SYNTHASE; DFR, DIHYDROFLAVONOL REDUCTASE; ANS, ANTHOCYANIDIN SYNTHASE; LAR, LEUCOANTHOCYANIDIN REDUCTASE; ANR, ANTHOCYANIDIN REDUCTASE; APX: ASCORBATE PEROXIDASE, GPX, GLUTATHIONE PEROXIDASE*. This figure has been created with BioRender.com.

Based on the results of this study, a conceptual model is proposed showing how constitutive levels of primary and specialized metabolites, H_2_O_2_, AsA, antioxidant enzymes, and defense-related genes prior to *B. cinerea* inoculation generate a series of reactions in strawberry leaves that led to the increased resistance of K1 leaves to *B. cinerea* (Figure 12). Summarized, first the defense-related genes *βGLU*, *JMT* and *AOS* showed a higher expression in K1 compared to T3 which could result in the increased resistance of K1. Second, the H_2_O_2_ content was lower in K1 compared to T3 and a negative correlation between the H_2_O_2_ content and plant resistance to *B. cinerea* was observed. Moreover, the activity of both antioxidant enzymes APX and GPX was higher in K1 and could contribute to the lower H_2_O_2_ content. Third, the precursors glucose, shikimic acid and phenylalanine of the phenylpropanoid pathway were more abundant in K1 compared to T3. Moreover, a higher expression of genes involved in the phenylpropanoid biosynthesis pathway was observed, which correlated with the higher levels of total phenolics and total flavonoids observed. As such, these compounds could contribute to plant defense directly or indirectly through H_2_O_2_ scavenging. Fourth, H_2_O_2_ can also be scavenged by high levels of ascorbic acid observed in K1 and generated from glucose using galactose as intermediate. Additionally, the higher citric acid content in K1 potentially inhibits H_2_O_2_ by inducing gene expression and enzyme activity of antioxidative enzymes (*APX, GPX*). Finally, the higher amount of sucrose in T3 may serve as a nutrient to promote *B. cinerea* growth, thus decreasing plant resistance. Further details of this model are discussed in detail below based on available literature.

#### Defense-Related Genes Involved in Strawberry Resistance to *Botrytis cinerea*

After *B. cinerea* inoculation, almost no induction of defense-related genes could be observed for both genotypes (Supplementary Figure 3). However, constitutive expression of the defense-related genes *βGLU, AOS,* and *JMT,* was higher in K1 compared to T3. *βGLU* can reduce pathogen virulence by degrading pathogen cell wall β-1,3-glucan. A higher expression of *βGLU* was observed in strawberries after treatment with β-aminobutyric acid treatment and resulted in increased resistance to *B. cinerea.* Moreover, after *B. cinerea* inoculation, *βGLU* was upregulated in strawberry fruit (Wang et al., 2016). AOS enzymes are involved in jasmonic acid biosynthesis (Farmer and Goossens, 2019), whereas JMT is responsible for converting JA to MeJA (Seo et al., 2001). The expression of *JMT* and *AOS* was higher in K1 compared to T3, suggesting that more JA and/or MeJA is present in K1, leading a stronger resistance to *B. cinerea* (Figure 4). The result is consistent with previous data reporting higher JA levels in a more resistant grape genotype compared to a susceptible genotype (Rahman et al., 2020). Moreover, a similar result was also found in Arabidopsis where overexpression of *JMT* resulted in increased resistance against *B. cinerea* (Seo et al., 2001). Overall, the higher expression of *βGLU*, *JMT* and *AOS* can contribute to the higher resistance of K1 to *B. cinerea*.

#### H_2_O_2_, Enzymatic Antioxidants and AsA Impact the Strawberry Resistance Against *Botrytis cinerea*

A hypersensitive response, leading to programmed cell death caused by an oxidative burst, is considered to be very important in restricting growth of biotrophic pathogens (Liu et al., 2019). Nevertheless, defense reactions effective against biotrophic pathogens are believed to increase susceptibility to necrotrophic pathogens since these micro-organisms can derive nutrients from dead cells (Lyon et al., 2007). For example, H_2_O_2_ levels correlated positively with *B. cinerea* growth and high H_2_O_2_ levels facilitate *B. cinerea* symptom development on Arabidopsis and strawberry leaves (Govrin and Levine, 2000; Meng et al., 2019). Moreover, a higher H_2_O_2_ content found in broad bean leaves after salicylic acid treatment under red light was associated with a higher susceptibility to *B. cinerea* (Khanam et al., 2005). In addition, *B. cinerea* infection can be suppressed by spraying antioxidants on plants (Elad, 1992). In grapes, low constitutive ROS production was also associated with a high level of resistance to *B. cinerea* (Rahman et al., 2020). Similar results were found in cucumber, where a lower accumulation of H_2_O_2_, O_2_^−^ was found in a resistant genotype compared to a susceptible genotype after *B. cinerea* inoculation (Yang et al., 2020). In this study, we observed that T3 leaves have a higher level of H_2_O_2_ compared to K1 and that there was a significant positive correlation between H_2_O_2_ content and symptom development (i.e., Lesion area) for both genotypes (Figure 5). We can, therefore, hypothesize that the higher H_2_O_2_ content promotes *B. cinerea* infection.

The low constitutive level of H_2_O_2_ observed in K1 compared to T3 could be attributed to several mechanisms, such as a higher activity of antioxidant enzymes, a higher content of phenolic and flavonoid compounds and AsA (Figures 5–10, 12). In this study, a significantly higher expression of genes encoding *GR*, *APX1*, *APX3* and *GPX*6_1 was observed in K1 compared to T3, while transcript levels of all other genes encoding antioxidant enzymes did not differ between the genotypes (Figure 6). Moreover, APX and GPX enzyme activities were also significantly higher in K1 compared to T3 (Figure 7). No significant differences were observed in *CAT* transcript levels and activity between the two genotypes, probably resulting from the higher affinity of APX for H_2_O_2_ than CAT (Pandey et al., 2017). Overall, these results suggest that K1 has more active H_2_O_2_ scavenging systems based on a higher *GPX6_3*, *GR*, *APX1* and *APX3* expression and higher GPX and APX activity compared to T3. Interestingly, treatment of plants, such as *Brassica juncea*, castor beans and sunflower, with citric acid increased the activity of SOD, CAT, APX, GPX and POD to reduce ROS content (Farid et al., 2017; Al Mahmud et al., 2018; Mallhi et al., 2019). Thus, the higher activity of APX and GPX in our study could be due to the higher citric acid content in K1 compared to T3 (Table 1; Figure 7). The importance of citric acid in increased plant resistance was further demonstrated by the negative correlation between citric acid content and lesion area (Table 1).

AsA is a well-known antioxidant that efficiently scavenges ROS (Horemans et al., 2000). A higher amount of AsA, APX activity and transcript levels and lower amounts of H_2_O_2_ were detected in K1 compared to T3 (Figures 5–8) which is in line with a previous report that AsA is participating in ROS scavenging *via* the action of APX (Das and Roychoudhury, 2014). The most important pathway to produce AsA is the L-galactose pathway which is generated from D-glucose via several intermediates, including L-galactose and fructose (Wheeler et al., 1998), higher concentrations of all these sugars were detected in K1 compared to T3 (Table 1; Supplementary Table 2; Figure 8). After AsA synthesis, APX oxidizes AsA to MDHA which spontaneously disproportionated into DHA. In turn, DHA can be reduced back to AsA by DHAR (Dell’Aglio and Mhamdi, 2020). Furthermore, previous research found a positive correlation between constitutive AsA levels and fruit resistance to *B. cinerea* in apple (Davey et al., 2007). Similarly, higher AsA levels were found on sun-exposed, less susceptible side of apple than the shaded side (Bui et al., 2019). In general, the higher levels of AsA and APX activities in K1 contribute to the decreased H_2_O_2_ content and increased tolerance to *B. cinerea*.

#### Phenolic Compounds and Their Biosynthesis Precursors and Intermediates Contribute to the Strawberry Resistance Against *Botrytis cinerea*

Phenolic compounds can have ROS scavenging activity and play an important role in plant defense (Pollastri and Tattini, 2011; Agati et al., 2012; Bose et al., 2014; Yang et al., 2018). Phenylpropanoids are synthesized via phenylalanine, derived from the shikimate pathway with shikimic acid as a central metabolite. In our study, K1 showed a higher level of phenolic compounds (shikimic acid, phenylalanine, total phenolics and flavonoids) compared to T3, and all compounds content showed a highly positive correlation with plant defense (Figures 9, 10; Table 1). The increased level of the total phenolics and flavonoids observed in K1 could be connected with the higher level of shikimic acid and phenylalanine and the higher expression of the genes involved in the phenylpropanoid pathway including *PAL, C4H, CCR, CHS, CHI, F3H, DFR, ANS, LAR,* and *ANR*. This result is in line with previous studies that unripe strawberry fruit with higher amounts of flavonoids and phenolics are less susceptible to *B. cinerea* (Di Venere et al., 1998; Puhl and Treutter, 2008; Aaby et al., 2012). Moreover, the higher levels of total phenolics after red light treatment also contributed to the increase in strawberry leaf tolerance to *B. cinerea* (Meng et al., 2019). Consequently, phenolic and flavonoid compounds in strawberry leaves play an important role as constitutive resistance mechanism against *B. cinerea* either directly or via scavenging H_2_O_2_.

#### Sucrose May Promote *Botrytis cinerea* Development in Strawberry

The increased accumulation of sucrose and total sugar content in T3 may promote *B. cinerea* development (Table 1; Supplementary Table 2). For example, sucrose was reported to promote *B. cinerea* growth and invasion in tomato leaves both *in vitro* and *in vivo.* Accordingly a tomato mutant with a lower total sugar content is less susceptible to *B. cinerea* (Asai et al., 2016; Courbier et al., 2020).

## Conclusion

In this study, we show that K1 is more resistant to *B. cinerea* than T3 and that the increased resistance is due to a combination of constitutive resistance mechanisms mainly linked to the antioxidative profile of K1. We observed a lower amount of H_2_O_2_ in leaves of K1 correlating with a higher activity of the antioxidant enzymes APX and GPX and a higher amount of non-enzymatic antioxidants (phenolic compounds, citric acid, and ascorbic acid). The increased level of phenolic compounds, caused by the higher expression of genes involved in the phenylpropanoid pathway and a higher level of the shikimic acid and phenylalanine, inhibited the build-up of high H_2_O_2_ levels could also directly contribute to plant resistance. Citric acid potentially reduced H_2_O_2_ levels by upregulating the activity of APX and GPX; similarly, high levels of AsA produced by increased levels of glucose and galactose might have resulted in reduced H_2_O_2_ content. In conclusion, our study reveals that the innate antioxidative profile of strawberry leaves plays a major role in the resistance of woodland strawberry leaves against *B. cinerea*.

## Data Availability Statement

The original contributions presented in the study are included in the article/Supplementary Material, further inquiries can be directed to the corresponding author.

## Author Contributions

YZ, BC, and MH designed the experiments. YZ performed all experiments, whereas KT was involved in the GC–MS experiments, and LV was involved in the disease assays, the quantification of fungal DNA, and the microscopic analysis. YZ analyzed the data and created all figures. YZ and BC wrote the manuscript with input from all co-authors. All authors read and approved the final manuscript.

## Funding

YZ has been supported by the China Scholarship Council at the KU Leuven (No. 201706990031). BC acknowledges financial support from the BelOrta chair (ITP-LSBEL1-O2010).

## Conflict of Interest

The authors declare that the research was conducted in the absence of any commercial or financial relationships that could be construed as a potential conflict of interest.

## Publisher’s Note

All claims expressed in this article are solely those of the authors and do not necessarily represent those of their affiliated organizations, or those of the publisher, the editors and the reviewers. Any product that may be evaluated in this article, or claim that may be made by its manufacturer, is not guaranteed or endorsed by the publisher.

## Footnotes

^1^ www.rstudio.com
