# Supplementary material for: Constitutive Defense Mechanisms Have a Major Role in the Resistance of Woodland Strawberry Leaves Against Botrytis cinerea
Source: Front Plant Sci. 2022 Jul 6;13:912667. doi: 10.3389/fpls.2022.912667 (PMC9298464; doi:10.3389/fpls.2022.912667)
Supplement: Supplementary file 1 [file Data_Sheet_1.docx]

**Supplementary materials:**

**Supplementary Text 1**

## **DNA extraction**

100 mg frozen sample was ground with mortar and pestle in the presence of liquid nitrogen and 10 mg polyvinylpolypyrrolidone (Sigma-Aldrich BVBA). The resulting powder was transferred to a sterile 2 mL Eppendorf tube containing 800 μL of pre-warmed (65 ℃) CTAB extraction buffer containing 15% cetyltrimethylammonium bromide (Carl Roth), 25% polyvinylpyrrolidone (Sigma Aldrich), 2 M Tris-HCL (Sigma Aldrich), 0.5 M EDTA (Carl Roth), 5 M sodium chloride (VWR) and 0.5% spermidine (Sigma Aldrich). Thereafter, 80 μL of 2-mercaptoethanol (Sigma Aldrich) and 2 glass beads were added and the samples were vortexed vigorously for 1 min and shaken at 5000 rpm for 2 min with a break of 15 s in between by Fastprep machine (Precellys 24 Tissue Homogenizer). Next, the samples were incubated at 65 ℃ for 30 min with 1400 rpm by Thermomixer (ThermoMixer C basic device without thermoblock 220-240V EU-plug) to lyse cells completely. Subsequently, an equal volume (800 μL) of chloroform: isoamyl alcohol (24:1) (VWR) was added and the tubes were vortexed vigorously for 1 min again, then the tubes were centrifuged at 15,700 x g for 15 min at 22 °C. The upper aqueous phase was transferred to a new 2 mL tube and this chloroform extraction step was repeated once. Approximately 400 µL of pre-cold isopropanol (Acros Organics BVBA) (-20 ℃) was added to the tube and the tubes were inverted 5 times to precipitate the nucleic acids and then put in the -80 ℃ freezer overnight. After that, the samples were centrifuged at 15,700 x g for 15 min at 4 ℃ and the supernatant was discarded. Next, pre-cold 70% ethanol (VWR) (-20 ℃) was added and the samples were vortexed for 2 min and then centrifuged again at 15,700 x g for 15 min at 4 ℃ and the supernatant was discarded. The pellet was air-dried and re-suspended in 200 µL of RNase-free-Milli-Q H_2_O. Then 2 µL 10 mg/mL RNAse A (Thermo Fisher Scientific) was added.

**Supplementary Text 2**

## **RNA extraction**

Grinding and addition of CTAB extraction buffer was performed as described for the DNA extraction method. Next, 80 μL of 2-mercaptoethanol (Sigma-Aldrich), 80 μl of 20 % N-lauroylsarcosine sodium salt solution (Sigma-Aldrich) and 2 glass beats were added and the samples were vortexed vigorously for 1 min and incubated at 65℃ for 10 min at 1300 rpm in a Thermomixer to lyse cells completely. Subsequently, an equal volume (800 μL) of chloroform:isoamyl alcohol (24:1) (VWR™) was added and the tubes were vortexed vigorously for 1 min again, and centrifuged at 15,700 x g for 15 min at 22 °C. After centrifugation, the upper phase was transferred to a new 1.5 mL tube. This step was repeated once. Approximately 600 µL from the upper phase was pipetted on a QIAshredder Mini Spin Column (RNeasy plant mini kit), and centrifuged at 8,000 x g for 1 min at 22 °C. Next, 400 µL of 100 % ethanol was added to the eluate, mixed immediately by pipetting and transfer to the RNeasy Mini Spin Column (RNeasy plant mini kit) and then centrifuged at 8,000 x g for 1 min at 22 °C. Next, the samples were washed twice with 500 µL of RPE buffer (RNeasy plant mini kit) and centrifuged again at 8,000 x g for 1 min at 22 °C. The RNeasy mini columns were centrifuged at 8,000 x g for 2 min to remove the ethanol before elution. The columns were transferred to a new 1.5 mL Eppendorf tube and 40 μL of water was added on the column to elute the RNA. The tubes were incubated for 5 min at room temperature and then centrifuged again at 8,000 x g for 1 min at 22 °C. RNA concentration and purity were measured using the NanoDrop (Thermo Fisher Scientific, Waltham, MA, USA).


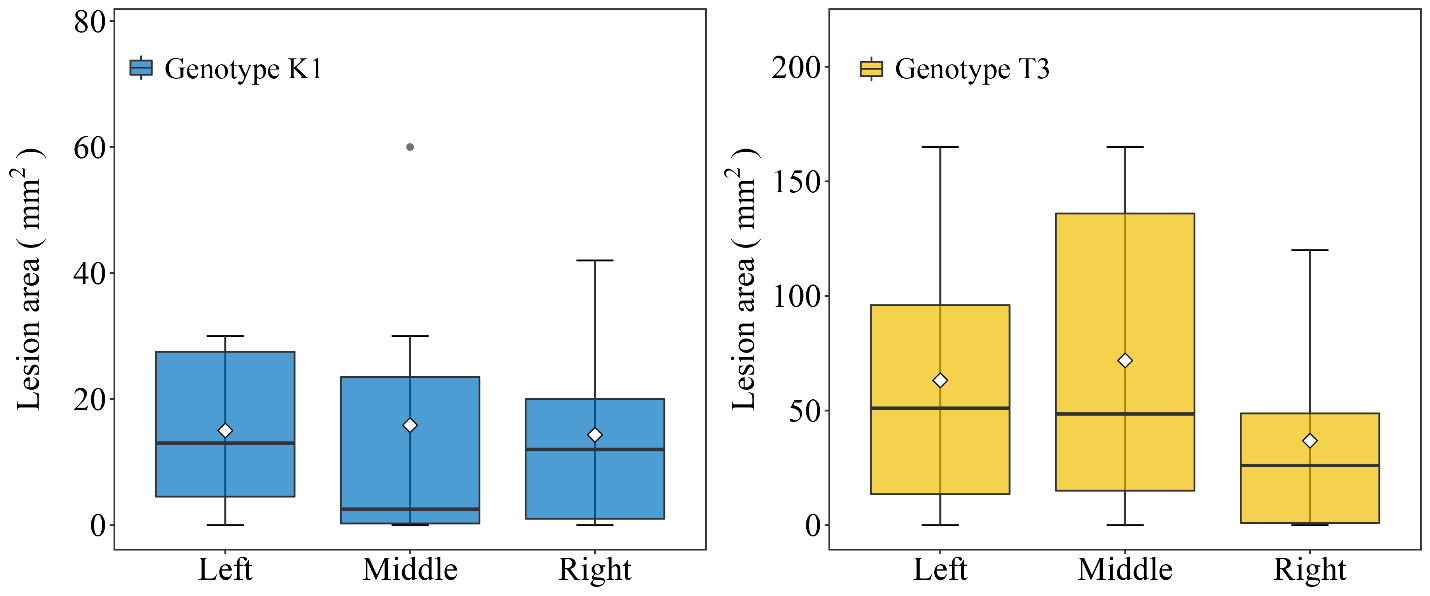


Supplementary Figure 1. Lesion area on strawberry leaflets of Kreuzkogel 1 (K1) and Tenno 3 (T3) at 5 days after *B. cinerea* inoculation. Trifoliate strawberry leaves were divided into three leaflets and used to evaluate the susceptibility against *B. cinerea*. Left: left leaflets; middle: middle leaflets; right: right leaflets. No significant differences between the three leaflets of the two genotypes were found by Kruskal-Wallis test at P < 0.05 (n=6). The box limits are the 25^th^ and 75^th^ percentile, the middle line in the individual boxes represents the median, the white point in the individual boxes marks the mean, whiskers extend to 1.5-fold the interquartile range of the 25^th^ and 75^th^ percentiles. The individual points represent the outliers.

A

B

C


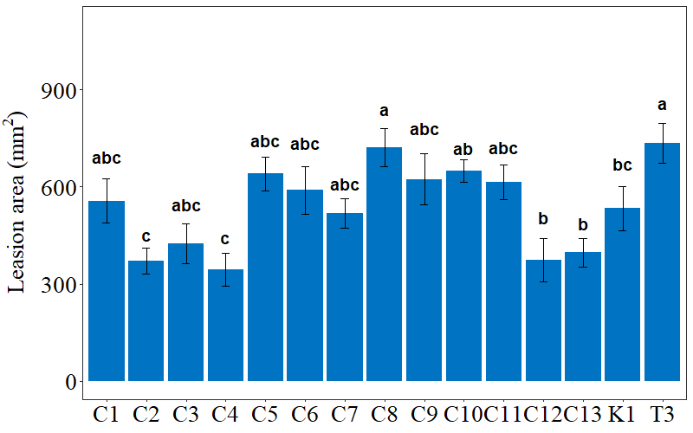

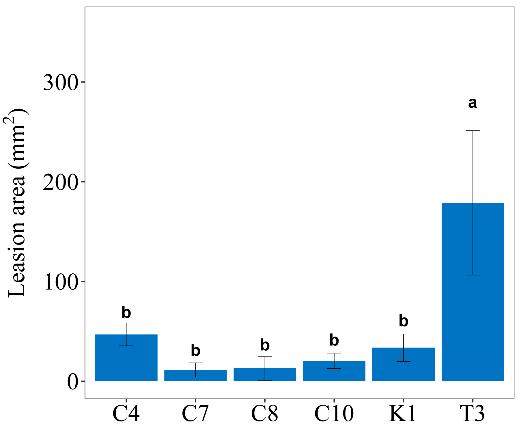

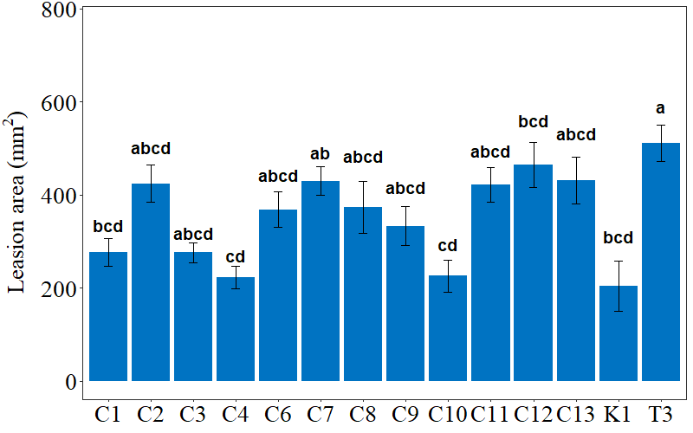


Supplementary Figure 2. Lesion area on strawberry leaves of different genotypes at 5 days after *B. cinerea* inoculation. (A) repeat 1, (B) repeat 2, (C) repeat 3. Different names on the X-axis mean different genotypes: C1: *F. vesca* ssp. bracteata BL-21-1, C2: *F. vesca* ssp. PI 602924; C3: *F. vesca* ssp. americana CFRA 954.001; C4: *F. vesca* ssp. bracteata British Colombia St. 98,04-4, C5: *F. vesca* ssp. vesca Böhmen; C6: *F. vesca* ssp. vesca Gulsrik, nahe Oslo, C7: *F. vesca* f. alba Moritzburg, C8: *F. vesca* ssp. vesca Rumänien, C9: *F. vesca* ssp. vesca Schottland, C10: *F. vesca* ssp. vesca Süd-Öland 1, C11: *F. vesca* ssp. vesca Versailles, C12: *F. vesca* ssp. semperflorens Rote Baron Solemacher, C13: *F. vesca* ssp vesca Hawaii 4, K1: *F. vesca* ssp. vesca Kreuzkogel 1, T3: *F. vesca* ssp. vesca Tenno 3. Different letters indicate significant differences between values by Kruskal-Wallis test at p < 0.05. Values are represented as means ± S.E. (n=10-15).


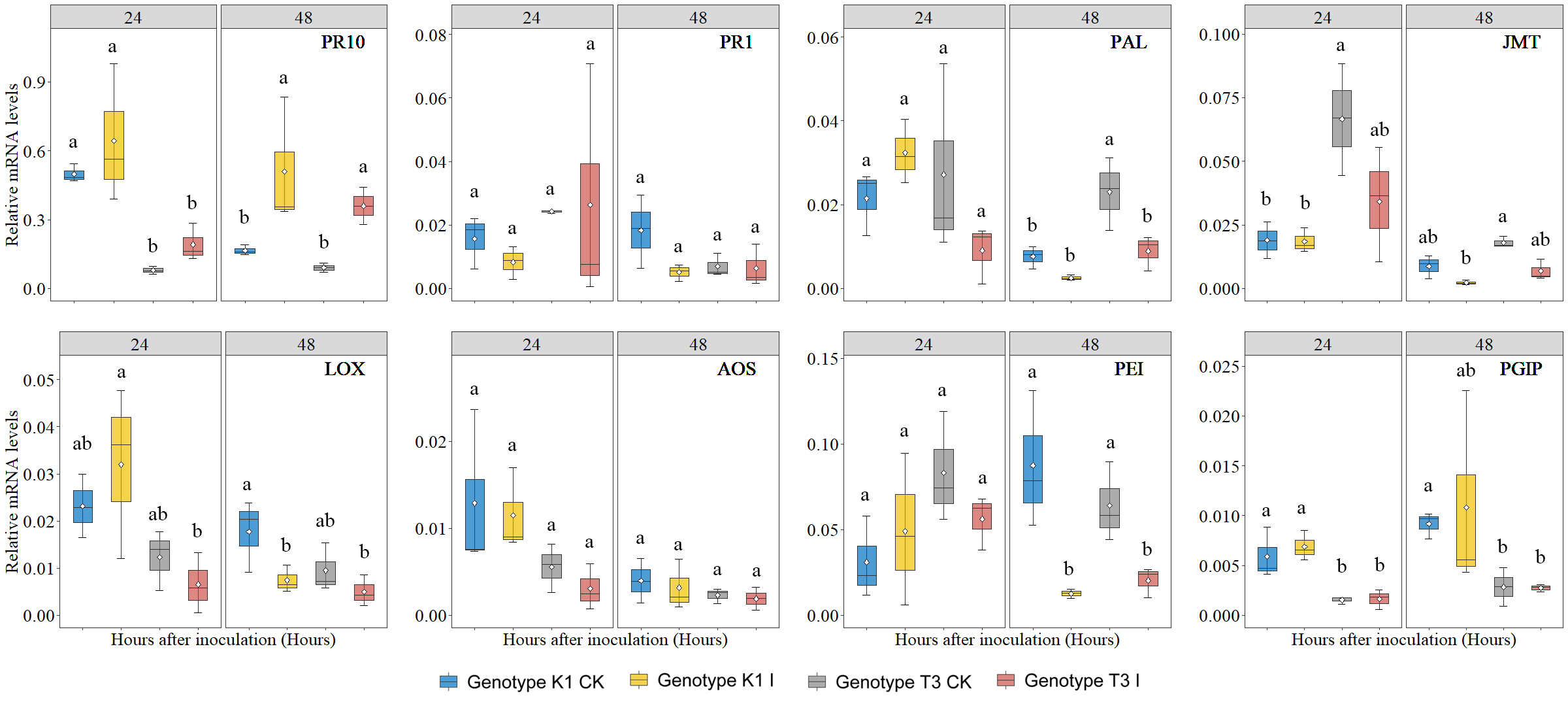


Supplementary Figure 3. Relative expression levels of eight defense-related genes in leaves of *F. vesca* ssp. vesca Kreuzkogel 1 (K1) and *F. vesca* ssp. vesca Tenno 3 (T3) at 24 and 48 hpi were measured via qRT-PCR. (CK: control group; I: inoculated group). All values were normalized to the expression level of the *H4* and *UBC9* housekeeping genes. Different letters indicate significant differences between values by Kruskal-Wallis test at p < 0.05 (n=3). The box limits are the 25^th^ and 75^th^ percentile, the middle line in the individual boxes represents the median, the white point in the individual boxes marks the mean, whiskers extend to 1.5-fold the interquartile range of the 25^th^ and 75^th^ percentiles. *PR10, PATHOGENESIS RELATED PROTEIN FAMILY 10*; *PR1, PATHOGENESIS RELATED PROTEIN FAMILY* 1; *PAL, PHENYLALANINE AMMONIA-LYASE;* *JMT, JASMONIC ACID CARBOXYL METHYLTRANSFERASE; LOX, LIPOXYGENASE;* A*OS, ALLENE OXIDE SYNTHASE; PEI, PECTINESTERASE INHIBITOR; PGIP, POLYGALACTURONASE INHIBITOR PROTEIN.*

**Supplementary Table1: Sequences of primers used for qPCR and qRT-PCR**

| Gene | Forward primer 5’→3’ | Reverse primer 5’→3’ | |
| --- | --- | --- | --- |
| *BcIGS* | GCTGTAATTTCAATGTGCAGAATC | GGAGCAACAATTAATCGCATTTC | |
| *FvPR1* | CTCACCCCAGGACTACCTCA | TAGAATGCACGAGGTTGCAG | |
| *FvLOX* | GGCAAATCCTCATCAATGCT | GCTCGGTGAATACCCAGTGT | |
| *FvPEI* | TGTGGACGTTGTGGTAGCTC | GCGCTTCAAGCTGTAATCCG | |
| *FvJMT* | GATTGGGATGGTGGTGCTGA | CGCCGCTGCTTATTGCTATG | |
| *FvPR10* | TCTCATCCCCAAGATTGCTC | CTGCCCTCACCAAAAGTGAT | |
| *FvAOS* | TGGGTTCTCTTCCAACTGA | CTCTGGTAGTCTTTCTTGACC | |
| *FvPGIP* | ATCTCACAGGTCCCATCCAG | GCTGAGGAAGTCAGGGACTG | |
| *FvPAL* | TGGTACTGCTGTTGGCTCTG | AGGCTTCCCCTGCATTACTT | |
| *FvPOD1* | AAACAAAACTGCCCACGAAC | GAAGGAGGCCCTTTGATTTC | |
| *FvPOD2* | GAAGAAGGGATTCGACCACA | GAGCTTGTCCGATGGAATGT | |
| *FvCAT* | TTCTCAGCCAATTCATGCAG | GACAACAAGGTGCTCCCAAT | |
| *Fvßglu* | TCATGTGAAGACCGGGACTC | TTGGGCTGTTTGTTAGGGGA | |
| *FvC4H* | CGCTATAGTCGTCGCAATCA | GTCAGTAAGGTTGCGGTGGT | |
| *Fv4CL* | TGATATTGACAAGGCGTTGAA | ACGACGAACAACTGAGTGACC | |
| *FvCCR* | CGTCGTCTTCACGTCTTCAA | GCAAAACTCGAGGTCACTCC | |
| *FvCHS* | AGTCTCAACGAGGCCTTCAA | CTCGTGGCTTCCAACTTCTC | |
| *FvCHI* | GCCGAGGAGTTGACAGAGTC | TTTCAATGGCTTTCGCTTCT | |
| *FvF3H* | AGAGAAGGAGGCATTGACGA | GAACGGTGATCCACGTCTTT | |
| *FvFLS* | CAACGAGGCCATATCCAAGT | TGGTAGAAGGTGGCCAAATC | |
| *FvDFR* | CTCTGGTCATCGGTCCATTT | ATGAGACTGGCAGAGGTCGT | |
| *FvANS* | GCTCGTCAACAAGGAGAAGG | TGTGCTGGATATGCTCGAAG | |
| *FvANR* | CCGACGAAAATGATTGGTCT | ATACTGCTGGGGATGTCTGG |  |
| *FvLAR* | CCGTTGGATCAGTTCCAGAT | TGCGACCAATTTTCTTCTCC |  |
| *FvNOX* | GGCAGAATACTGGGCGAATA | TTCCAGGGAAGACGGTGTAG |  |
| *FvRbohA* | CTCCATGCTGGGAATCATCT | CGGTTTATGCGTTCCAAAGT |  |
| *FvCuSOD1* | ATGGATGTGCCAGGAAAGTC | TTCCAGATCTCGGCATTTTT |  |
| *FvCuSOD2* | TGATGCTGTATTGCCAGAGC | GGTTGCACCCAGAATCCTAA |  |
| *FvCu_ZnSOD1* | AGGAAGAATCATGGGCCTCT | GAGCGGATTGAAGTGAGGTC |  |
| *FvCu_ZnSOD2* | CCATTTGCATGAGTTTGGTG | GATTGTTGCCTCTGCCTCTC |  |
| *FvFeSOD* | AAGCCTCCTCCATACCCACT | GCTCAGTTCCTTCGATTTGC |  |
| *FvMnSOD* | TCACCGGATTAGGTTTCCAG | GTGCTTCTGGTGGTGGATTT |  |
| *FvGR* | TTGCAATAGTTGGTGGTGGA | TTTGCTCAGCGAGAAATCCT |  |
| *FvAPX1* | GGTCAGTGGGGCTTATTTGA | ATCAGGACCCAAGAATGCAG |  |
| *FvAPX3* | GACTGAAAATCGCGGTTGAT | TAGGTGGACCTCCAGTGACC |  |
| *FvUBC9* | GCAACATGTGGCATACAAGG | TGGCATTTCTTTTCCGAATC |  |
| *FvH4* | ATCACCAAGCCTGCTATTCG | GAGAGCATACACGACGTCCA |  |

**Supplementary Table 2: Other metabolites from GC-MS detected for K1 and T3**

| **Metabolites** | **RT (min)** | **Genotype K1** | | | **Genotype T3** | | |
| --- | --- | --- | --- | --- | --- | --- | --- |
|  |  | **Content (mg/g)** | ***R^d^*** | ***P^e^*** | **Content (mg/g)** | ***R^d^*** | ***P^e^*** |
| Ribose ^a^ | 7.39 | 0.0007 ± 0.0000 | -0.09 | 0.71 | 0.0010 ± 0.0001 ^ns^ | -0.05 | 0.86 |
| Arabitol ^a^ | 7.63 | 0.0041 ± 0.0002 | -0.20 | 0.44 | 0.0042 ± 0.0009 ^ns^ | -0.07 | 0.79 |
| Fucose ^a^ | 7.78 | 0.0087 ± 0.0008 | -0.19 | 0.45 | 0.0051 ± 0.0005 ^***^ | 0.07 | 0.79 |
| Myo-inositol ^a^ | 9.49 | 1.0404 ± 0.0302 | 0.07 | 0.79 | 1.0030 ±0.0954 ^ns^ | -0.10 | 0.71 |
| Quininic acid ^b^ | 8.36 | 4.9443 ± 0.7440 | -0.18 | 0.47 | 3.4382 ± 0.6445 ^**^ | -0.32 | 0.22 |
| Alanine ^a^ | 16.65 | 0.0365 ± 0.0038 | 0.05 | 0.86 | 0.0294 ± 0.0048 ^ns^ | -0.41 | 0.11 |
| Glycine ^a^ | 17.10 | 0.1425 ± 0.0243 | -0.04 | 0.87 | 0.0291 ± 0.0053 ^ns^ | -0.30 | 0.27 |
| GABA ^bc^ | 20.72 | 0.0210 ± 0.0029 | -0.15 | 0.54 | 0.0183 ± 0.0027 ^ns^ | -0.50 | 0.05 |
| Succinate ^a^ | 20.78 | 0.1114 ± 0.0070 | -0.10 | 0.70 | 0.1888 ± 0.0308 ^ns^ | -0.32 | 0.23 |
| Fumaric ^b^ | 21.29 | 0.0201 ± 0.0024 | -0.41 | 0.10 | 0.0135 ± 0.0005 ^**^ | -0.00 | 0.98 |
| Threonine ^a^ | 25.16 | 0.0615 ± 0.0053 | 0.34 | 0.16 | 0.1057 ± 0.0199 ^ns^ | -0.42 | 0.10 |
| Malic acid ^a^ | 26.57 | 0.4885 ± 0.0374 | -0.16 | 0.52 | 0.7946 ± 0.1295 ^ns^ | -0.22 | 0.42 |
| Asparagine ^a^ | 29.35 | 0.0066 ± 0.0012 | -0.00 | 0.99 | 0.0233 ± 0.0085 ^ns^ | -0.16 | 0.55 |
| Glutamine ^a^ | 30.99 | 0.0109 ± 0.0023 | -0.15 | 0.55 | 0.0068 ± 0.0021 ^ns^ | -0.33 | 0.22 |
| Tyrosine ^a^ | 33.75 | 0.0034 ± 0.0005 | -0.37 | 0.13 | 0.0037 ± 0.0003 ^ns^ | -0.33 | 0.21 |
| Tryptophan ^a^ | 34.26 | 0.0033 ± 0.0005 | 0.06 | 0.83 | 0.0110 ± 0.0043 ^ns^ | -0.31 | 0.24 |
| Total sugars ^af^ | -- | 4.033 ± 0.151 | 0.65 | 0 | 4.831 ± 0.216 ^**^ | 0.69 | 0 |

Significant differences between two genotypes are represented by*( p < 0.05), ** (p < 0.01), *** (p < 0.001) ****( p < 0.0001).

a Wilcox test was used to check the significant difference between two groups.

b t test was used to check the significant difference between two groups.

c GABA: Gamma aminobutyric acid

d Pearson’s correlation coefficient r between lesion area which measured on strawberry left leaflets at 5 days after *B. cinerea* inoculation and other metabolites in strawberry middle and right leaflets for K1 and T3, respectively, without *B. cinerea* inoculation.

e Probabilities of significance of linear models relating lesion area which measured on strawberry left leaflets at 5 days after *B. cinerea* inoculation to other metabolites in strawberry middle and right leaflets for K1 and T3, respectively, without *B. cinerea* inoculation.

f The total sugars content equal to the sum of fructose content, glucose content and sucrose content.
